# Fast and general tests of genetic interaction for genome-wide association studies

**DOI:** 10.1371/journal.pcbi.1005556

**Published:** 2017-06-06

**Authors:** Mattias Frånberg, Rona J. Strawbridge, Anders Hamsten, PROCARDIS consortium, Ulf de Faire, Jens Lagergren, Bengt Sennblad

**Affiliations:** 1 Cardiovascular Medicine Unit, Department of Medicine, Solna, Karolinska Institutet, Stockholm, Sweden; 2 Department of Numerical Analysis and Computer Science, Stockholm University, Stockholm, Sweden; 3 Science for Life Laboratory, Stockholm, Sweden; 4 Division of Cardiovascular Epidemiology, Institute of Environmental Medicine, Karolinska Institutet, Stockholm, Sweden; 5 Department of Cardiology, Karolinska University Hospital, Stockholm, Sweden; 6 The School of Computer Science and Communications, KTH Royal Institute of Technology, Stockholm, Sweden; 7 Swedish e-science Research Center (SeRC), Stockholm, Sweden; Microsoft Research, UNITED STATES

## Abstract

A complex disease has, by definition, multiple genetic causes. In theory, these causes could be identified individually, but their identification will likely benefit from informed use of anticipated interactions between causes. In addition, characterizing and understanding interactions must be considered key to revealing the etiology of any complex disease. Large-scale collaborative efforts are now paving the way for comprehensive studies of interaction. As a consequence, there is a need for methods with a computational efficiency sufficient for modern data sets as well as for improvements of statistical accuracy and power. Another issue is that, currently, the relation between different methods for interaction inference is in many cases not transparent, complicating the comparison and interpretation of results between different interaction studies. In this paper we present computationally efficient tests of interaction for the complete family of generalized linear models (GLMs). The tests can be applied for inference of single or multiple interaction parameters, but we show, by simulation, that jointly testing the full set of interaction parameters yields superior power and control of false positive rate. Based on these tests we also describe how to combine results from multiple independent studies of interaction in a meta-analysis. We investigate the impact of several assumptions commonly made when modeling interactions. We also show that, across the important class of models with a full set of interaction parameters, jointly testing the interaction parameters yields identical results. Further, we apply our method to genetic data for cardiovascular disease. This allowed us to identify a putative interaction involved in Lp(a) plasma levels between two ‘tag’ variants in the LPA locus (*p* = 2.42 ⋅ 10^−09^) as well as replicate the interaction (*p* = 6.97 ⋅ 10^−07^). Finally, our meta-analysis method is used in a small (*N* = 16,181) study of interactions in myocardial infarction.

## Author summary

Interaction between organic molecules forms the basis of all biological systems. The availability of high-throughput genotyping and sequencing platforms enables us to cost-effectively genotype a large number of individuals. For sufficiently large datasets it is possible to reconstruct the genetic dependencies that underlie complex traits and diseases. However, there is a need for efficient statistical methodologies that can tackle the large sample size and computational resources required to study interaction. In this work we provide theory that reduces the required computational resources, and enable multiple research groups to effectively combine their results.

## Introduction

Large data sets are vital to counter low statistical power due to low allele frequency, small effect sizes, and multiple testing. This has driven the GWAS field towards more collaborative efforts as well as meta-analyses. Fortunately, there exists a standardized statistical methodology that allows for reliability and comparability between different studies. In contrast, in association studies aiming at identifying interactions, or epistasis, there are multiple competing methodologies with unclear relationships. As a consequence, collaborative GWAS efforts have almost exclusively focused on single variant associations.

Conceptually, interactions in association studies are generated when multiple genetic variants affect the dynamic, non-linear and inter-connected networks that underlie complex traits [1]. Candidate-based medical genetic studies established early that interaction may play an important role in complex diseases [2–7]. Consequently, over the last five years, substantial attention has been devoted to resolving the statistical and computational problems associated with large-scale studies of interactions [8–15]. However, the formulation of these methods is still not easily compared, and crucial differences between the underlying interaction models are not always transparent. This makes comparability between studies still a major concern and hampers opportunities for meta-analysis. There is, therefore, a need to harmonize interaction models and investigate the implications of their assumptions.

From a statistical point of view it is non-trivial to define interaction [16] and it can often be unclear how different assumptions, e.g., on the main effect of each variant, affect the definition of interaction. There has been some work aimed at a standardized description of interaction models [17, 18]. However, these studies specifically targeted a class of models, in which any two models are related by a linear one-to-one transformation, which limits their applicability. In this paper, we present a more general framework that enables modeling and interpretation of genetic interactions in the context of any generalized linear model (GLM). This can be applied to, in principle, any type of outcome (e.g., continuous, binary, factor or count phentoypes) or model of interaction. We show how this new formulation can be used to analyze the relation between various interaction models.

Multiple tests for interaction have been proposed for case-control data. However, these tests typically depend on strong assumptions about the main effects, marginal effects or LD to reduce the computational complexity [15, 19, 20]. Recently Yu et al. introduced a closed-form Wald test restricted to a specific parameterization of the logistic regression model [21]. Here, we introduce a general class of computationally efficient Wald tests, that enables analysis of case-control traits, quantitative traits, and in fact any trait modeled by a member in the exponential family. More importantly, these tests allow for any combination of parameterization and link function to be used, that is, it can be applied to all the models considered here. Moreover, we show that our Wald test can be applied in large-scale meta-analyses.

A major complication in interpreting interactions is that they are inferred relative to a link function. This function determines the parameter subspace that belongs to the null model and is, in practice, unknown. Consequently, mis-specification of the link function causes an inflated error rate that increases with sample size, which cannot be resolved by replication in a separate cohort. Here we address this issue by testing interactions using a family of link functions. Specifically we use two families of link functions that has been proposed previously [22, 23]. We also show that the previously suggested goodness-of-link test [24] is not appropriate for joint testing of interaction parameters.

We implement these new tests in a GLM-based analysis tool for both case-control and quantitative data. We investigate the impact of different parameterizations on both the false positive rate and the statistical power. We finally apply our Wald tests in two genome-wide interaction analyses. Firstly, we study a continuous phenotype, Lp(a), in the PROCARDIS cohort. Secondly, we perform a meta-analysis of myocardial infarction by combining results from the PROCARDIS cohort and the Myocardial Infarction Genetics Consortium cohort.

## Results

### Theory

#### Introduction to generalized linear models in genetics

In this subsection, we introduce the theory of GLMs and their application to genetic data. We then describe how a so-called parameterization can be used to incorporate genetic assumptions about a single variant. Finally, we describe how a multi-variant parameterization can be constructed from several single variant parameterizations.

In this work, we use GLMs to describe the relation between predictor variables, which here typically are genotypes, and an outcome variable, that is, the phenotype. For each individual *i*, *Y*_*i*_ is a measured phenotype and ***x***_*i*_ is a vector of predictor variables. The observed phenotype *y*_*i*_ is modeled by its expected value *E*[*Y*_*i*_ ∣ ***X***_*i*_] = *μ*_*i*_ along with a distribution from the exponential family that captures the stochastic variation around *μ*_*i*_. We write *y*_*i*_ ∼ *f*(*μ*_*i*_) and refer to *f* as the *dispersion distribution*. The expected value *μ*_*i*_ is in turn related to the *linear predictor* *ψ*(***x***_*i*_)***β*** by *g*(*μ*_*i*_) = *ψ*(***x***_*i*_)***β***, where *g* is the *link function*, *ψ* is an encoding of the predictor variables, and ***β*** is a vector of parameters. A combination of *ψ* and ***β*** is called a *parameterization*. The parameters are generally estimated according to the maximum likelihood principle by applying the iteratively reweighted least squares algorithm. The commonly used linear regression is a special case of GLMs, obtained by using the identity link function (*g*(*μ*_*i*_) = *μ*_*i*_) and the Normal dispersion distribution. Moreover, logistic regression is a GLM with the logit link function ($g\left(\mu \right)=\log\left(\frac{\mu }{1-\mu }\right)$) and a Binomial dispersion distribution.

As our focus here is interactions, we restrict the predictor variables ***x***_*i*_ to be genotypes. Because the set of possible genotypes for a set of variants are finite, and in practice often fewer than the number of individuals, many individuals will have identical ***x***_*i*_. It is therefore convenient to define a model that is indexed by an enumeration of the genotypes *h* instead of by individuals *i*. In this notation, ***p***_*h*_ is the *design vector* of the genotype that corresponds to *h*, and, for an individual *i* with genotype *h*, *μ*_*h*_, the expected value of *y*_*i*_, is modeled by *g*(*μ*_*h*_) = ***p***_*h*_***β***. Here *h* can be the genotype of either a single variant or multiple variants. Furthermore, we can define a design matrix *P*, so that ***p***_*h*_ are the rows of *P*; the complete relationship between the mean levels of the genotypes and the parameterization can then expressed *g*(***μ***) = *P****β***.

In general, we will call any model where the number of parameters equals the number of unique design vectors a *saturated* model, otherwise it is called *unsaturated*. Additionally, we will call a model *full* if the number of unique design vectors equals the number of possible genotypes, otherwise it is called *constrained*. A design matrix that recodes genotypes into dominant and recessive states is an example of a constrained model.

*Single-variant models.* We first introduce a notation for the parameterization for a single variant and, in the next section, we extend this notation to multiple variants. We let ***β*** = (*α*, *β*_1_, *β*_2_)^*T*^, where *α* is referred to as the reference level, and *β*_1_ and *β*_2_ are deviations from the reference (the ^*T*^ indicates the transpose, i.e., ***β*** is a column vector). For example, if ***p***_*h*_ = (1, 2, 0) then *g*(*μ*_*h*_) = *α* + 2*β*_1_. For the single variant case, let *A* be the most frequent haploid genotype in the population considered, and *a* be the less frequent. We have three possible diploid genotypes {*AA*, *Aa*, *aa*} that are enumerated by *h* ∈ {0, 1, 2}. We now describe the parameterizations corresponding to the most common single variant genetic models.

- Saturated full parameterizations
  - *G*—Genotypic. Let *β*_1_ be the mean difference in phenotype between the reference and the heterozygote, and *β*_2_ be the mean difference in phenotype between the reference and the minor homozygote. Then the genotypic can be expressed as follows:
    $$g\left(\begin{array}{c} \mu_{0}\\ \mu_{1}\\ \mu_{2} \end{array}\right)=\left(\begin{array}{ccc} 1 & 0 & 0\\ 1 & 1 & 0\\ 1 & 0 & 1 \end{array}\right)\left(\begin{array}{c} \alpha \\ \beta_{1}\\ \beta_{2} \end{array}\right).$$
  - *AD*—Additive/deviation. Letting *β*_1_ denote the additive component, and *β*_2_ denote the deviation from additivity, this can be expressed as follows:
    $$g\left(\begin{array}{c} \mu_{0}\\ \mu_{1}\\ \mu_{2} \end{array}\right)=\left(\begin{array}{ccc} 1 & 0 & 0\\ 1 & 1 & 1\\ 1 & 2 & 0 \end{array}\right)\left(\begin{array}{c} \alpha \\ \beta_{1}\\ \beta_{2} \end{array}\right).$$
- Saturated constrained parameterizations
  - *R*—Recessive. This model assumes that the effect allele has a recessive effect:
    $$g\left(\begin{array}{c} \mu_{0}\\ \mu_{1}\\ \mu_{2} \end{array}\right)=\left(\begin{array}{cc} 1 & 0\\ 1 & 0\\ 1 & 1 \end{array}\right)\left(\begin{array}{c} \alpha \\ \beta_{1} \end{array}\right).$$
  - *D*—Dominant. This model assumes that the effect allele has a dominant effect:
    $$g\left(\begin{array}{c} \mu_{0}\\ \mu_{1}\\ \mu_{2} \end{array}\right)=\left(\begin{array}{cc} 1 & 0\\ 1 & 1\\ 1 & 1 \end{array}\right)\left(\begin{array}{c} \alpha \\ \beta_{1} \end{array}\right).$$
  - *H*—Heterozygote. This model assumes an heterozygote advantage effect:
    $$g\left(\begin{array}{c} \mu_{0}\\ \mu_{1}\\ \mu_{2} \end{array}\right)=\left(\begin{array}{cc} 1 & 0\\ 1 & 1\\ 1 & 0 \end{array}\right)\left(\begin{array}{c} \alpha \\ \beta_{1} \end{array}\right).$$
- Unsaturated full parameterizations
  - *A*—Additive. This model assumes that only additive effects are present:
    $$g\left(\begin{array}{c} \mu_{0}\\ \mu_{1}\\ \mu_{2} \end{array}\right)=\left(\begin{array}{cc} 1 & 0\\ 1 & 1\\ 1 & 2 \end{array}\right)\left(\begin{array}{c} \alpha \\ \beta_{1} \end{array}\right).$$

We note that *G* alternatively could be perceived as either a Heterozygote/deviation, *HD*, or a Recessive/deviation, *RD*.

**Fig 1:**
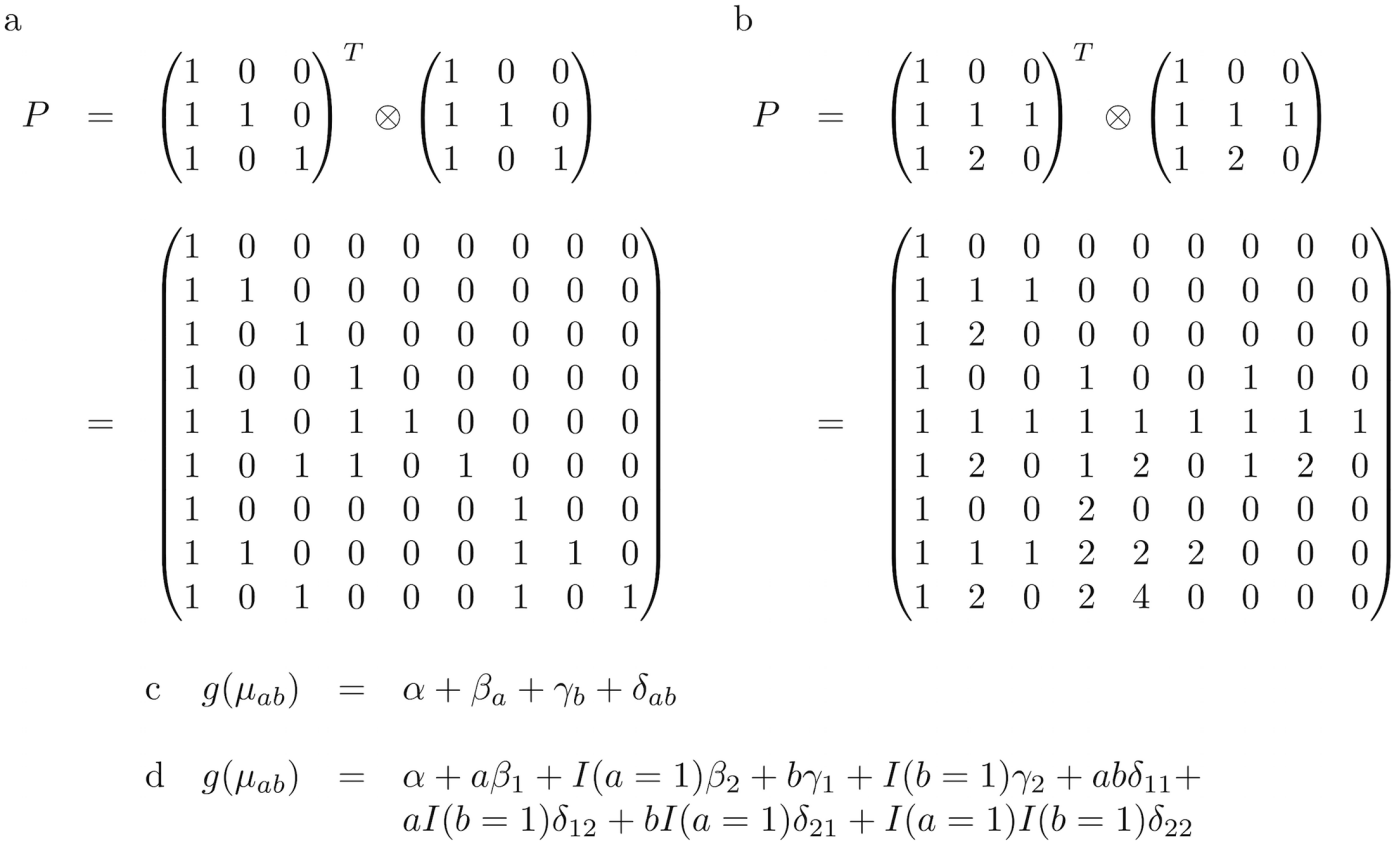
Two examples of the construction of bi-variant interaction models as the Kronecker product (⊗) of two uni-variate models. (a) The construction of the *P* matrix of the *G* × *G* model, (b) The construction of the *P* matrix of the *AD* × *AD* model, (c) the *G* × *G* model in bi-variant notation, (d) the *AD* × *AD* model in bi-variant notation. For both models, the resulting parameter vector ***β*** = (*α*, *β*_1_, *β*_2_, *γ*_1_, *δ*_11_, *δ*_12_, *γ*_2_, *δ*_21_, *δ*_22_). In c) and d) *a* ∈ {0, 1, 2} is the genotype for the first variant and *b* ∈ {0, 1, 2} is the genotype for the second variant, implicitly *β*_0_ = *γ*_0_ = *δ*_0*_ = δ_*0_ = 0, and, lastly, *I*(*x*) is an indicator function taking the value 1 if *x* is true and 0 otherwise.

*From single to multi-variant models.* We will now show how a multi-variant parameterization with relevant interaction parameters can be obtained from a set of single variant parameterizations. This provides a intuitive way to construct higher order interaction parameterizations. Thereafter, we provide two examples of the construction of bi-variant models (see Fig 1).

A multi-variant parameterization can be constructed from single variant parameterizations using the Kronecker-product (⊗) [17]. Given a design matrix, *P*^(*i*)^, for each variant *i* ∈ {1…*k*}, in which each row parameterizes the corresponding genotype, then the multi-variant design matrix, *P*, for these variants is

$$\begin{array}{c} P=P^{\left(k\right)}⊗P^{\left(k-1\right)}⊗\ldots ⊗P^{\left(1\right)}. \end{array}$$ (1)

The parameters ***β*** for the multi-variant model can similarly be constructed by taking the Kronecker-product of the parameter vectors

$$\begin{array}{c} \beta =\beta^{\left(k\right)}⊗\beta^{\left(k-1\right)}⊗\ldots ⊗\beta^{\left(1\right)}, \end{array}$$ (2)

where, in each Kronecker operation ***β***^(*k*)^ ⊗ ***β***^(*k*−1)^ (*i*) the intercept has been replaced by 1 in all parameter vectors ***β***^(⋅)^ on the right hand side of Eq (2), (*ii*) the first element of the resulting vector ***β*** on the left hand side of Eq (2) is the new intercept, and (*iii*) any product between two different parameters is replaced by a new parameter that represents interaction. The number of factors in each element represents the order of interaction and factors of order 1 are the main effects. The matrix *P* and the parameters ***β*** are now related to the mean values by

$$\begin{array}{c} g\left(\mu \right)=P\beta \Rightarrow \mu =g^{-1}\left(P\beta \right)\;. \end{array}$$

We will below mainly focus on bi-variant models resulting from applying Eqs (1) and (2) to two uni-variant models, say *M*_*i*_ and *M*_*j*_, of those enumerated above in Section Introduction to generalized linear models in genetics. We denote the resulting bi-variant model *M*_*i*_ × *M*_*j*_. We detail the construction of *G* × *G* and *AD* × *AD* in Fig 1. Notice that the Kronecker-product of two saturated single-variant models yield a saturated bi-variant model.

This concludes our description of the GLM components used to model genotype-phenotype association. Using this uniform GLM framework, we formulate, in the next section, efficient tests for interaction applicable to very general families of GLMs, allowing any number of variants and a wide variety of phenotypes and link functions. Moreover, in Section Relating different GLMs, we use this framework in a comparative analysis of major GLM families with respect to differences in parameterization, link function and dispersion distribution.

#### Fast estimation and testing of interaction in generalized linear models

In this section, we first derive closed-form estimates of the parameters of a GLM and the corresponding covariance matrix for any saturated parameterization. We then build on this to design an computationally efficient test for interaction. A complete discussion and proofs can be found in S1 Text. We generalize these results to unsaturated models using a two-step estimation procedure. Finally, we show how the Wald tests can be used in a meta-analysis.

*A general Wald test for saturated models.* We first describe how to estimate the parameters. Let *N* be a diagonal matrix in which the diagonal element *N*_*hh*_ is the number of individuals with the genotype corresponding to *h*. Let ***t*** be a vector in which element *t*_*h*_ contains the sum of the phenotypic values of individuals with the genotype corresponding to *h*. Maximum likelihood estimates of ***β*** is then obtained by

$$\begin{array}{c} \beta =P^{-1}g\left(N^{-1}t\right) \end{array}$$ (3)

where the inverse of *P* exists because *P* is a full rank square matrix when the parameterization is saturated (see S1 Text). The time complexity of this estimation is, assuming that matrix inversion can be performed in cubic time, $O(3^{T}n+3^{3T}+3^{2T})$ where *T* is the number of variants in the model and *n* is the number of samples. The first term is for computing the sufficient statistics, the second for the matrix inversion of *P*, and the third for computing the product between the transformed sufficient statistics and *P*^−1^. Here, *P*^−1^ only needs to be computed once, and *T* is typically small (*T* = 2 in this article), so that the dominant time complexity is linear in the number of samples O(n).

The covariance matrix of the saturated full parameterization can be similarly derived, and has the following simple form

$$\begin{array}{c} C={\left(P^{T}\right)}^{-1}I^{-1}P^{-1} \end{array}$$

where I is the Fisher information matrix. The inverse of the Fisher information matrix is a diagonal matrix with elements on the form

$$\begin{array}{c} I_{hh}^{-1}=\frac{\phi v_{h}g^{\prime }{\left(\mu_{h}\right)}^{2}}{n_{h}} \end{array}$$

where *v*_*h*_ = *Var*(*y*_*h*_ ∣ *p*_*h*_, *ϕ* = 1), which can be calculated from the specific dispersion distribution that is used, $g^{\prime }\left({\hat{\mu }}_{h}\right)$ is the derivative of the link function evaluated at ${\hat{\mu }}_{h}$ (the estimate of *μ*_*h*_), *n*_*h*_ is the number of samples with genotype *h*, and *ϕ* is the dispersion parameter of the GLM (see S1 Text).

The general test for interaction in association analysis evaluates whether the relevant interaction parameters are significantly different from zero. There are three asymptotically equivalent tests that can be used to test the interaction parameters: the score test, the Wald test, and the explicit likelihood ratio test (LRT). The score test requires estimation only under the null hypothesis, the Wald test requires estimation under the alternative hypothesis, and the standard likelihood ratio test under both. For saturated GLMs, Eq (3) provides an efficient parameter estimation under the alternative hypothesis, and, consequently, we base our test for interaction on the multivariate Wald test. Let $\hat{\delta }$ denote the vector of estimated interaction parameters, and ${\hat{C}}_{\delta }$ denote the sub-matrix of the estimated covariance matrix that corresponds to the interaction parameters. The Wald test statistic is a quadratic form in $\hat{\delta }$ constructed using the estimated covariance matrix, and the test is defined as

$$\begin{array}{c} W\left(\hat{\delta }\right)={\hat{\delta }}^{T}{\hat{C}}_{\delta }^{-1}\hat{\delta }. \end{array}$$

This Wald test requires computing the inverse of a typically small covariance matrix *C*_*δ*_. Consequently, the total time complexity of estimation and testing is $O(3^{3T}n+3^{3T}+3^{2T})$. Again, as *T* is small, the dominant time complexity is linear in the number of samples *n*. Our Wald test is applicable to any saturated GLM, and contains, as a special case, the previously described Wald test for logistic regression [21].

The score test and the LRT additionally requires estimation of ***β*** under the null model of no interaction, for which no closed form expression exists. Hence, in this case, we need to resort to the slower iteratively reweighted least squares (IRLS) algorithm. The average time complexity for estimation and testing based on the IRLS algorithm is $O(\left(3^{T}n+3^{3T}\right)A\left(n\right))$ where *A*(*n*) is the expected number of iterations until convergence. Since the time complexity for computing the actual test statistics is similar to that of the Wald test, these two tests will have a theoretical complexity that is dominated by a factor that is linear in *n*. The practical difference is shown in S1 Fig, in which the Wald test derived in this paper gives a speedup of around 20-100 compared to the likelihood ratio test implemented using the ILRS algorithm. The speed of the Wald test also compares favorably with the less general interaction test in the highly optimized Plink 1.9 software, we note that this test is limited to an *A* × *A* model with linear or logistic link function.

*Unsaturated models.* For an unsaturated model the design matrix *P* is no longer full rank, and for parameter estimation one usually must resort to an iterative algorithm. We develop a two-step closed-form estimation, in which we first transform the model to a convenient saturated model (e.g., *G* × *G*), by extending *P* and ***β*** with corresponding columns and parameters, respectively. We estimate all parameters in this model, and then back-transform them to the unsaturated model.

Let *P****β*** be the parameterization of the unsaturated model, and *P*_*S*_***β***_*S*_ be the parameterization of a corresponding saturated model. For convenience of notation let $S=P_{S}^{-1}P$; we then obtain the following relationship between the parameters of the saturated and unsaturated model: ***β***_*S*_ = *S****β***. Given an estimate of the covariance matrix *C*_*S*_ of the saturated parameters we can estimate ***β*** and the corresponding covariance matrix *C* by

$$\begin{array}{c} \hat{\beta }={\left(S^{T}{\hat{C}}_{S}^{-1}S\right)}^{-1}S^{T}{\hat{C}}_{S}^{-1}{\hat{\beta }}_{S} \end{array}$$

and

$$\begin{array}{c} \hat{C}=S^{T}{\hat{C}}_{S}^{-1}S. \end{array}$$

The idea underlying these estimators is that we can view the estimated parameters of the saturated model $\hat{\beta_{S}}$ as an outcome of a multivariate Normal distribution with covariance matrix *C*_*S*_ and mean *S****β***. Maximum likelihood estimation of ***β*** in this model leads to the equations above (this can be equivalently viewed as a generalized least squares estimation). We show in S1 Text that this leads to a consistent estimator.

The time complexity involves estimating the saturated model, transforming these estimates into estimates for the unsaturated model, and computing the final Wald test statistic. This time complexity is bounded by the estimation of the saturated model, so the total time complexity is still $O(3^{T}n+3^{3T}+3^{2T})$. For the score and LRT tests the time complexity is, assuming one parameter per variant, $O(\left(2^{T}n+2^{3T}\right)A\left(n\right))$.

*Fixed effect meta-analysis of multivariate Wald tests.* We now describe how to combine association results, based on the Wald test, from multiple studies to perform a meta-analysis. Let ${\hat{\beta }}_{i}$ denote the vector of estimated coefficients from study *i* and ${\hat{C}}_{i}$ the corresponding covariance matrix. The combined $\hat{\beta }$ across studies is estimated with

$$\begin{array}{c} \hat{\beta }=\sum_{k=1}^{M}{\left(\sum_{i=1}^{M}{\hat{C}}_{i}^{-1}\right)}^{-1}{\hat{C}}_{k}^{-1}{\hat{\beta }}_{k} \end{array}$$

and the combined covariance matrix with

$$\begin{array}{c} \hat{C}={\left(\sum_{i=1}^{M}{\hat{C}}_{i}^{-1}\right)}^{-1}. \end{array}$$

The combined Wald statistic across studies is then $\hat{\beta }{\hat{C}}^{-1}\hat{\beta }$ which follows a *χ*^2^-distribution with four degrees of freedom. Of note, each covariance matrix is commonly small and the time for computing the combined covariance matrix is shorter than the time for estimating each individual covariance matrix.

A practical problem for large-scale meta-analysis is that the result files from the interaction analysis of each study will be very large, typically in the order of 1 Terabyte. We suggest one possible solution to this issue. The analysis can be split into two stages. In the first stage, each study reports all variant pairs below some p-value threshold. A reduced set of candidate variant pairs are then created by taking the union of the significant pairs over all studies. In the second stage, all studies perform a second analysis of the reduced set of variant pairs. Finally, meta-analysis is performed on the result from the second stage. This effectively limits the storage space required, but may miss variant pairs with intermediate effects in all studies.

#### Relating different GLMs

In this section, we demonstrate some aspects of how interaction models can be related to each other within the GLM framework.

*Linear reparameterizations of saturated models are equivalent.* We first show that a certain class of parameterizations are equivalent with respect to the Wald test, providing some insight to when two parameterizations are identical in terms of the inference of epistasis.

We start by demonstrating an important property of the Wald test; if two parameterizations with interaction parameters ***δ*** and ***δ***′, respectively, are *linear* transformations of each other, i.e. ***δ***′ = *B****δ***, then the corresponding Wald tests are equivalent,

$$\begin{array}{c} W\left(\delta^{\prime }\right)=\delta^{\prime }C_{\delta^{\prime }}^{-1}\delta^{\prime }=B^{T}\delta {\left(B^{T}C_{\delta }B\right)}^{-1}B\delta =\delta C_{\delta }^{-1}\delta =W\left(\delta \right). \end{array}$$

We observe that an important corollary of this is that a joint test, i.e. testing all interaction parameters simultaneously, for the *G* × *G* and *AD* × *AD* parameterizations is equivalent. In general, any joint test of interaction for saturated reparameterizations, will have identical results. However, this is not true if parameterizations are non-linear transformations of each other, i.e. if they use different link functions.

Of note, the degrees of freedom can be reduced if some of the genotypes contains very few samples. We can then avoid estimating the interaction parameters for these cells and adjust the degrees of freedom accordingly. This could, however, cause the GLMs to be non-equivalent in terms of the joint likelihood, and an interaction in one parameterization may not be reflected in another.

*Relating parameter estimates.* We show above that two saturated full GLM reparameterizations are equivalent in terms of the joint Wald test. However, different saturated full parameterizations may emphasize different interaction components, which potentially reflect different biological mechanisms. We now investigate this further.

The relationship between two saturated full parameterizations *P****β*** and *Q****β***′ is

$$\begin{array}{c} \beta =P^{-1}Q\beta^{\prime } \end{array}$$

Importantly, the interaction parameters of ***β*** are a function solely of the interaction parameters of ***β***′. This is in contrast to the main effect parameters of ***β***, which may depend on both main effect and interaction parameters of ***β***′. The relationship between the interaction parameters of *G* × *G* and *AD* × *AD* is,

$$\begin{array}{ccc} \delta_{11}^{G\times G} & = & \delta_{11}^{AD\times AD}+\delta_{12}^{AD\times AD}+\delta_{21}^{AD\times AD}+\delta_{22}^{AD\times AD}\\ \delta_{12}^{G\times G} & = & 2\delta_{11}^{AD\times AD}+2\delta_{21}^{AD\times AD}\\ \delta_{21}^{G\times G} & = & 2\delta_{11}^{AD\times AD}+2\delta_{12}^{AD\times AD}\\ \delta_{22}^{G\times G} & = & 4\delta_{11}^{AD\times AD}, \end{array}$$

and

$$\begin{array}{ccc} \delta_{11}^{AD\times AD} & = & 1/4\cdot \delta_{22}^{G\times G}\\ \delta_{12}^{AD\times AD} & = & 1/2\cdot \delta_{22}^{G\times G}-1/4\cdot \delta_{21}^{G\times G}\\ \delta_{21}^{AD\times AD} & = & 1/2\cdot \delta_{22}^{G\times G}-1/4\cdot \delta_{12}^{G\times G}\\ \delta_{22}^{AD\times AD} & = & \delta_{11}^{G\times G}-1/2\cdot \delta_{12}^{G\times G}-1/2\cdot \delta_{21}^{G\times G}+1/4\cdot \delta_{22}^{G\times G}. \end{array}$$

We observe that the two parameterizations distribute the total interaction effect differently over the interaction parameters. For example, a single double homozygote interaction is apparent in *G* × *G*: *δ*^*G*×*G*^ = {0, 0, 0, 1}, but not in *AD* × *AD*: *δ*^*AD*×*AD*^ = {0.25, 0.5, 0.5, 0.25}. Conversely, a single additive-additive interaction is apparent in *AD* × *AD*: *δ*^*AD*×*AD*^ = {1, 0, 0, 0}, but not in *G* × *G*: *δ*^*G*×*G*^ = {1, 2, 2, 4}. We note that, for certain questions, exploring multiple parameterizations would be beneficial to allow the formulation of most-parsimonious constrained parameterizations, or to provide hypotheses on possible biological mechanisms for inferred interactions.

When performing the corresponding investigation of parameter relations of a non-saturated full parameterization, *P****β***, to another parameterization, *Q****β***′, we find that the interaction parameters of ***β*** are no longer guaranteed to be a function of the ***β***′ interaction parameters alone, but can depend on its main effects as well. More generally, constrained/non-saturated models reduce the number of interaction parameters (and sometimes also the main effect parameters) of saturated full parameterizations, either by constraining some parameters to be zero or to be functions of other parameters; some examples of the relation between the *G* × *G* parameterization and selected constrained or unsaturated parameterizations are given in Table 1.

**Table 1:** Relation between *G* × *G* interaction parameters and those of selected constrained or unsaturated models.

Model	*G* × *G* parameters			
	*δ*_11_	*δ*_12_	*δ*_21_	*δ*_22_
*A* × *A*	*δ*	2*δ*	2*δ*	4*δ*
*R* × *R*	0	0	0	*δ*
*D* × *D*	*δ*	*δ*	*δ*	*δ*
*R* × *D*	0	0	*δ*	*δ*
*H* × *H*	*δ*	0	0	0

The link function determines how fast the phenotype mean, *μ*, changes with the genotype. The choice among major classes of link function is further discussed, below, in Section Relating different GLMs and we will here focus on understanding the effect of a small change in the link function has on the parameters ***β***. If the link function *g*(***μ***) is perturbed to *g*(***μ***) + ***ϵ*** (while keeping the design matrix *P* constant), we have the following approximate relationship between the parameters in the two models,

$$\begin{array}{c} \beta \approx \beta^{\prime }+P^{-1}\text{diag}\left(\epsilon \right)\nabla_{\mu }g\left(g^{-1}\left(P\beta^{\prime }\right)\right) \end{array}$$

where ∇_*x*_ *f*(*x*) denotes the gradient of *f*(*x*) with respect to *x*. This means that the incorrect link function will introduce a bias in the parameter estimates, and the magnitude of that bias will depend on the rate of change of the inverse of the link function, as well as on the design matrix (some examples are given in S1 Table).

Finally, the dispersion distribution models the variation around the phenotype mean *μ* (given by the link function and the parameterization). However, for a given data set, the dispersion distribution is seldom varied because different dispersion distributions are often tightly connected to specific types of phenotypes (see examples in S2 Table). In particular, if, for a given dispersion distribution, *f*, with (shape) parameter *θ*, there exists a link function *g*(*μ*) such that *θ* = *μ*, then *g*(*μ*) is called the *canonical* link function of *f* (S2 Table).

*Examining the effect of different link functions.* In the previous sections, we observed that a small perturbation of the link function may bias the parameter estimates, and, for general changes in the link function the corresponding Wald tests may not be equivalent. This suggests that some interactions may not be consistent between link functions. In fact, it is well-known that, under some circumstances, interactions inferred with one link function might be absent when another link function [25, 26] is used. Loftus [25] referred to these interactions as “uninterpretable”, because although they represent an interaction we cannot be sure unless we know the true underlying link function. However, Loftus also defined a class of “interpretable” interactions whose inference is invariant of the link function.

To make a robust statement of the existence of a particular interaction, we must therefore determine whether the interaction seems to be invariant of the link function. One previously proposed link function test investigates systematic bias in the residuals [24]. The test is called a goodness-of-link test and is constructed by first fitting GLM with the canonical link, to obtain a first estimate of $\hat{\mu }$, and then fitting the same GLM with $g{\left(\hat{\mu }\right)}^{2}$ as an additional covariate to investigate non-linearity. However, for saturated GLMs, the goodness-of-link test becomes over-parameterized, and therefore meaningless, because there are no degrees of freedom left after fitting parameters to accommodate $g{\left(\hat{\mu }\right)}^{2}$ (it does work in the special case of unsaturated GLMs).

We will instead use another approach that tests interaction over multiple link functions in a family of link functions, parameterized by some parameter λ [22, 23]; we will refer to this test as the *link family* test. Many such families have been suggested, but the most important property is to be able to model functions increasing either faster or slower than the canonical link function. For simplicity we have selected the following families for the Normal and Binomial dispersion distributions, respectively:

$$\begin{array}{ccc} g\left(\mu \right)=\left\{\begin{array}{cc} \log(\mu ) & \lambda =0\\ \frac{\mu^{\lambda }-1}{\lambda } & \lambda \leq 1\\ {\left(1+\mu (2-\lambda )\right)}^{1/(2-\lambda )} & \lambda >1\\ e^{\mu } & \lambda =2 \end{array}\right. & \; & g\left(\mu \right)=\left\{\begin{array}{cc} \log\left(\frac{\mu }{1-\mu }\right) & \lambda =0\\ \frac{{\left(\frac{\mu }{1-\mu }\right)}^{\lambda }-1}{\lambda } & \text{otherwise} \end{array}\right. \end{array}$$

The first one is a symmetric version of the Box-Cox family of power transforms that lies between the log-link (λ = 0) and the *exp*-link (λ = 2). It also contains the identity link as a special case λ = 1. The second family, the Pregibon family, was suggested for studies of link functions for Binomial outcomes which contains the *logit*-link (λ = 0) as a special case [24]. These families are by no means a complete representation of all possible links, but serve as an attempt to broaden the family of null models.

Evaluation of link function invariance is a computer-intensive approach as it requires re-analyses while stepping through different values of λ to identify any critical link function. In practice, we therefore apply this test on significant pairs, only, as an *a posteriori* measure of link appropriateness.

In the following sections we investigate how the false positive rate and statistical power is affected by different assumptions on the genetic effects. In both cases, we evaluate the following parameterizations: the *G* × *G joint*, *AD* × *AD joint*, *G* × *G separate*, *A* × *A*, and *R*/*D* × *R*/*D* tests (described below). We then apply the *G* × *G joint* test to two biological data sets to demonstrate the efficacy and plausibility of our method. In the following sections, we will (unless it is clear from context) discriminate between a *generative model* referring to the model from which data is generated and a *test model*, which is used in the statistical test. For details on data generation, please see Materials and methods.

### Evaluated strategies for testing interactions

We will now introduce five different strategies for testing interactions. We will use these strategies together with the Wald test described in Section Fast estimation and testing of interaction in generalized linear models, above, in our investigations of statistical power and false positive rate.

The first three are based on two different saturated parameterizations: *G* × *G* and *AD* × *AD* (Fig 1). The *G* × *G* test models is the standard approach for regression on discrete variables in the GLM literature, the *AD* × *AD* test model corresponds to the *F*_∞_ *model* discussed by [17] (the careful reader will notice that, compared to [17], we have changed the coding of the second column of *AD* from (0, −1, 1) to (0, 1, 2) to allow for easy comparison with *G*). A parameterization that features multiple interaction parameters can be tested either *jointly* or *separately*. Let the interaction parameter vector be denoted by ***δ*** = {*δ*_11_, *δ*_21_, *δ*_12_, *δ*_22_, }. A *joint* test evaluates the hypothesis that all interaction parameters are zero ***δ*** = 0 and a *separate* test evaluates the hypothesis that one or more interaction parameters are zero ∪_*h*_{*δ*_*h*_ = 0}; notice for the separate test that, while each test has a lower degree of freedom, the multiple testing burden will increase by a factor of 4. In our first two strategies, the interaction parameters are tested by a joint test and we will refer to these tests as *G* × *G joint* and *AD* × *AD joint*, respectively, while the third strategy is based on a separate test and the *G* × *G* parameterization, which we refer to as *G* × *G separate*.

For the next strategy, we introduce a non-saturated parameterization that assumes that the genotypic effect is completely additive in the number of minor alleles, and thereby ignores possible deviations. This corresponds to the Kronecker product of two additive-encoded uni-variant GLMs (*A* ⊗ *A*). This model is often written

$$\begin{array}{c} g\left(\mu_{ab}\right)=\alpha +a\beta_{1}+b\gamma_{1}+ab\delta_{11} \end{array}$$

This is a much more restricted test model and the interaction is now represented by a single parameter *δ*_11_ instead of four as in the previous models. We refer to this test as the *A* × *A* test.

The last strategy, which has been applied in multiple studies of interactions [9, 27], is to first encode the genotypes into binary variables according to dominance or recessiveness. These binary variables are then analyzed separately in an interaction test. This encoding corresponds to the (saturated) parameterizations *D* × *D*, *R* × *D*, *D* × *R* and *R* × *R*:

$$\begin{array}{ccc} g(\mu_{ab}) & = & \alpha +I(a\geq 1)\beta +I(b\geq 1)\gamma +I(a\geq 1)I(b\geq 1)\delta \\ g(\mu_{ab}) & = & \alpha +I(a\geq 1)\beta +I(b=2)\gamma +I(a\geq 1)I(b=2)\delta \\ g(\mu_{ab}) & = & \alpha +I(a=2)\beta +I(b\geq 1)\gamma +I(a=2)I(b\geq 1)\delta \\ g(\mu_{ab}) & = & \alpha +I(a=2)\beta +I(b=2)\gamma +I(a=2)I(b=2)\delta \end{array}$$

In each GLM, interaction is measured by a single parameter *δ*. In analogy with separate testing, we evaluate if *δ* in any of the test models deviate from zero, and the multiple testing burden will increase by a factor of 4. We refer to this family of tests as *R*/*D* × *R*/*D*.

### Error rates under model-misspecification

**Fig 2:**
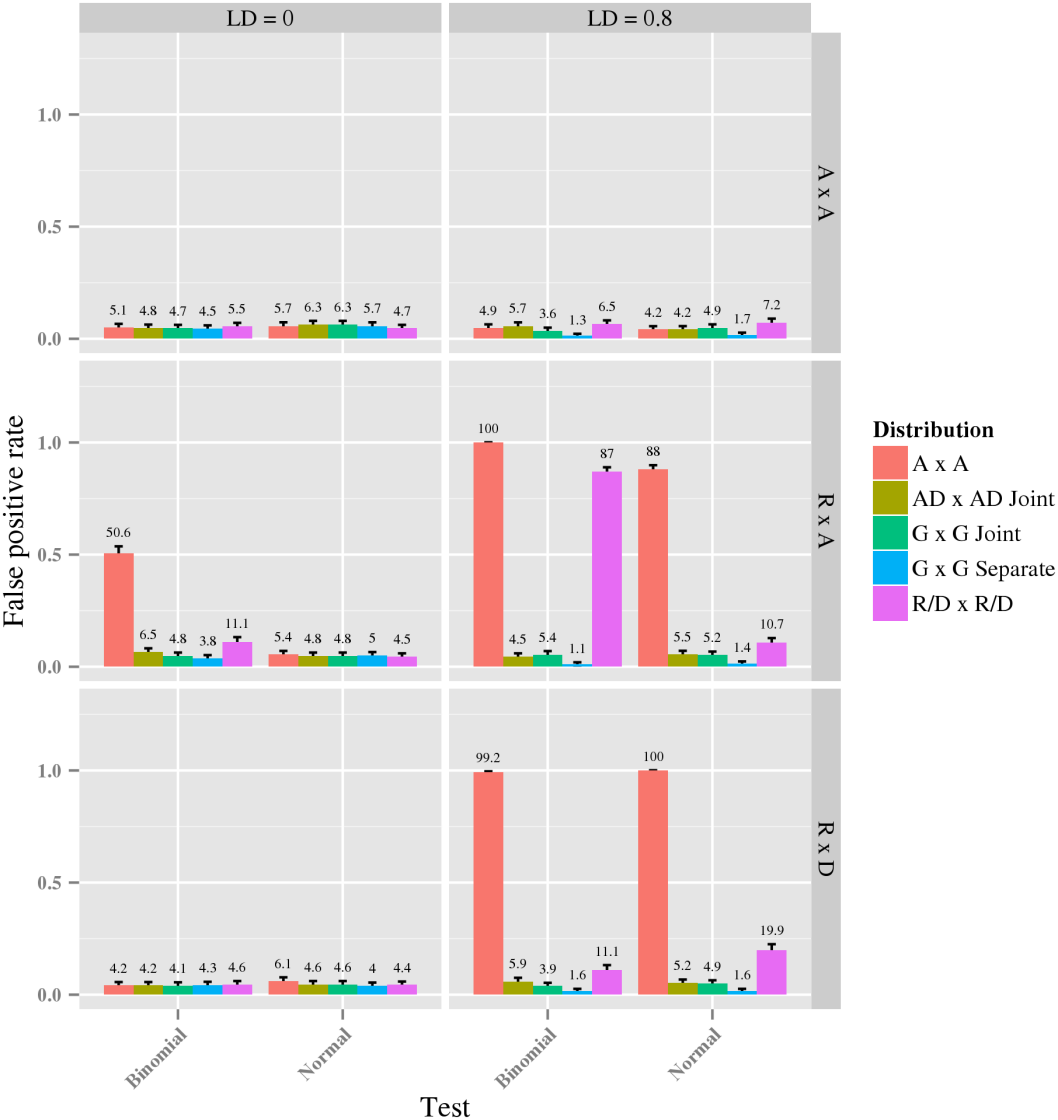
The estimated false positive rate for each test under six different generative models. Data was generated with all interaction parameters set to zero in this plot. For each subplot, the *y*-axis indicates the estimated false positive rate and the x-axis indicates the dispersion distribution. The rows correspond to null generative models under three different parameterizations: *A* × *A*, *R* × *A* and *R* × *D*. The columns correspond to two cases, no LD and an LD of 0.8 measured with Lewontin’s D. The colored bars refer to different interaction tests used, as indicated by the legend next to the plots.

We performed two experiments to investigate how potential model misspecifications affect the false positive rate (FPR) for the interaction tests described previously. In the first experiment we generated synthetic data from (null) models with no interaction, but where some assumptions of the evaluated tests fail (specifically, the presence of recessive and dominance for the *A* × *A* test and the presence of an additive component for the *R*/*D* × *R*/*D* test); we also tested whether linkage disequilibrium (LD) affects the FPR. The results in Fig 2 show that all tests controlled the error rate when there is no LD for a Normal dispersion distribution. However, for the Binomial dispersion distribution, the error rate of the *A* × *A* and *R*/*D* × *R*/*D* tests was inflated by the presence of dominant and recessive inheritance patterns. A second source of errors is LD, and the *A* × *A* test had a strongly inflated error rate when data was generated from *R* × *A* and *R* × *D* under both dispersion distributions. Moreover, also the *R*/*D* × *R*/*D* parameterization had an inflated error rate for all generative models under LD. The error rate was highest for data generated from *R* × *A* and a Binomial dispersion distribution. In general, the *G* × *G joint*, *G* × *G separate* and *AD* × *AD joint* tests are safe to use in the presence of LD, whereas the other tests must be treated with caution.

**Fig 3:**
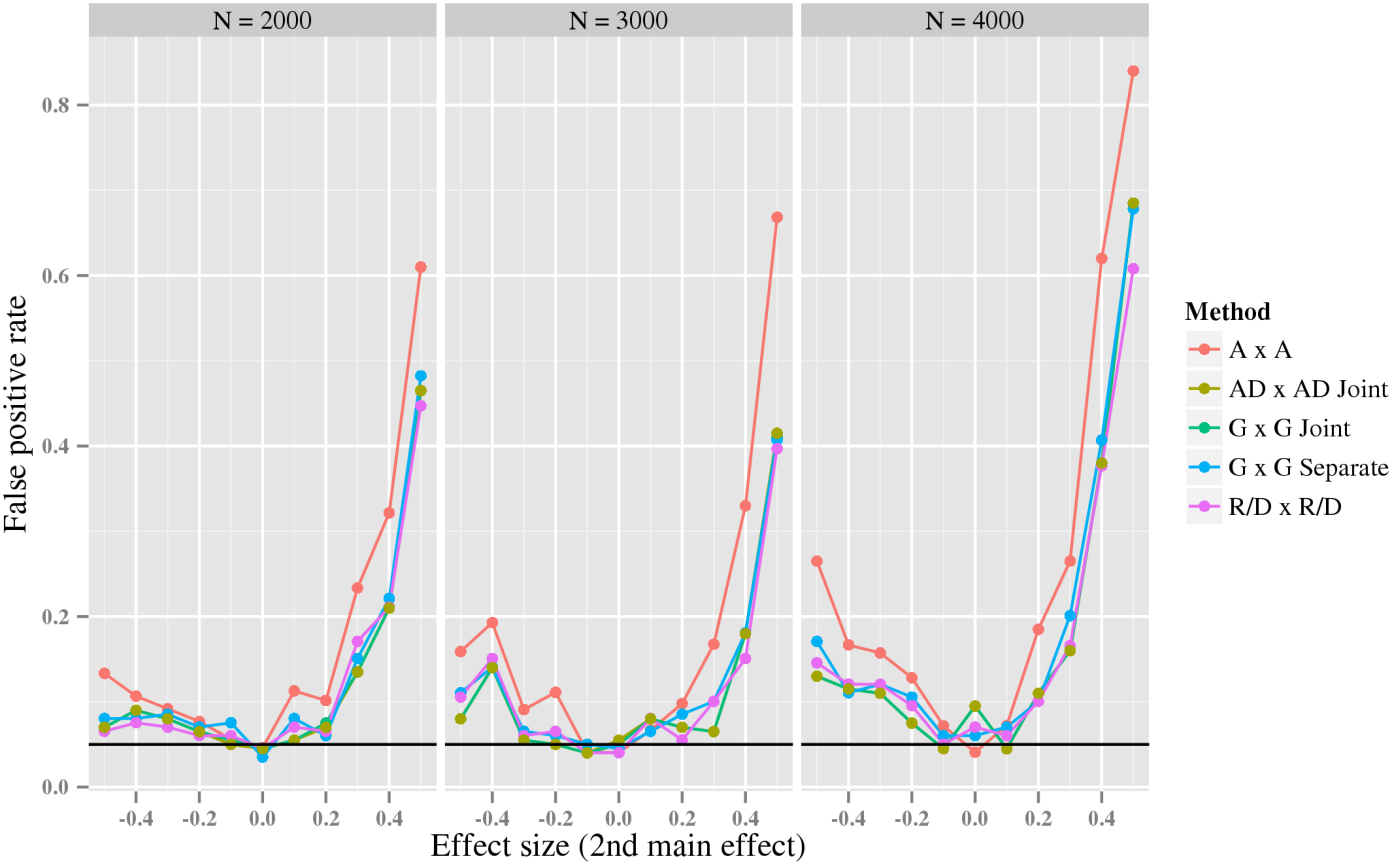
The estimated false positive rate under link function misspecification as a function of the second main effect. The x-axis indicates the effect size of the second main effect under a *A* × *A* generative model, while the y-axis indicates the estimated false positive rate. The colored lines correspond to the estimated false positive rate using different interaction tests, as indicated by the legend next to the plots. The black line is the desired 0.05 level. The data was simulated using the *log* link function, and tested using an identity link.

In the second experiment, we investigated the impact of link function misspecification on the FPR. As discussed in Section Relating different GLMs, it is known that interactions inferred with one link function might be absent when another link function [25, 26] is used, and vice versa. Here we test how often link misspecification introduce “false interactions”. We generated data from the *A* × *A* model with the log link function, whereas we performed each test using the identity link function. We measured the FPR as a function of the main effect of the second variant, while the main effect of the first variant is constant. Notice that when the second main effect is zero, the phenotype depends only on the first variant and, thus, no inflation of “false interactions” is expected. The results (Fig 3) show that all tests were affected similarly by link misspecification, and the false positive rate quickly increased with the second main effect’s deviation from zero; this effect was slightly more pronounced for the *A* × *A* test. The effect became more pronounced as the sample size increased. This demonstrates, as previously predicted [25], that use of an erroneous link function may lead to false inference of interaction.

### Statistical power

Designing a convincing and realistic experiment for measuring the expected statistical power is challenging. Ideally, we want to generate data from a biologically relevant model. However, neither the effect sizes, nor the structure of such a model are known. In addition, there is a large number of possible interaction models, even for single pair interactions where we have nine model parameters and two allele frequencies. In an attempt to balance efficiency and exhaustive exploration of this parameter space, we, perform two separate experiments. The first considers data generated from a small set of common interaction models, and a second, that considers data generated from a larger set of models, to give an overall picture of how different tests perform in relation to each other.

**Fig 4:**
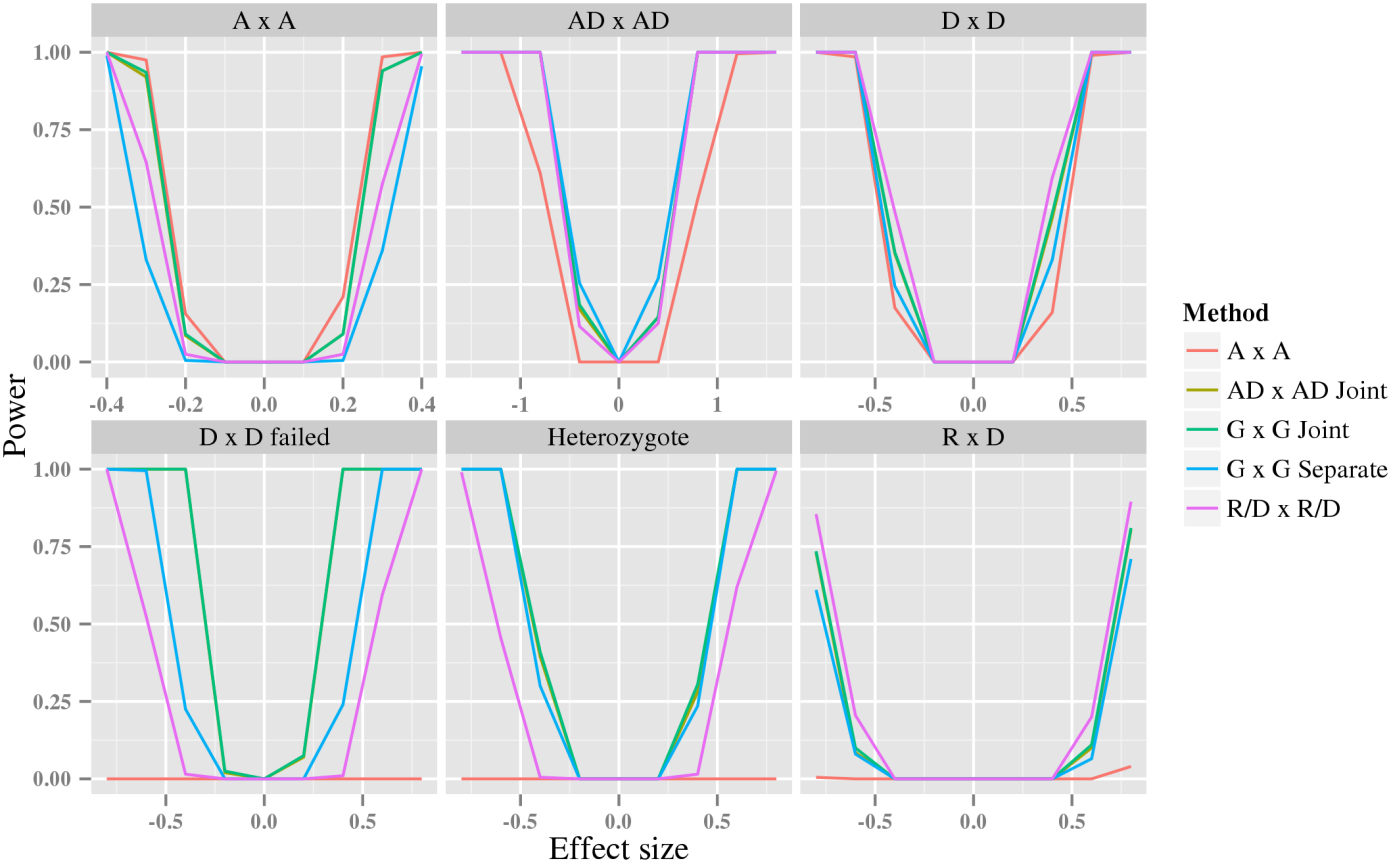
The statistical power of different testing strategies. The y-axes is the estimated statistical power, while the x-axis represents the effect size specific to each generative model: *δ*_11_ for the *A* × *A* model, *δ*_11_ = *δ*_12_ = *δ*_21_ = *δ*_22_ for the *AD* × *AD*, *δ*_11_ = *δ*_12_ = *δ*_21_ = *δ*_22_ for the *D* × *D* model, *δ*_11_ = *δ*_22_ = −*δ*_12_ = −*δ*_21_ for the *D* × *D* failed model, *δ*_11_ for the heterozygote model, and *δ*_12_ = *δ*_22_ for the *R* × *D* model. The sample size was 4000 and the minor allele frequency for both variants was 0.3. Notice that the line for the *AD* × *AD joint* test in all plots coincide with, and is hidden by, the line for the *G* × *G joint* test.

In the first experiment, we estimated the statistical power on data generated from the *A* × *A*, *A* × *A*^*failed*^, *D* × *D*, *D* × *D*^*failed*^, *H* × *H*, and *R* × *D* GLMs. The *A* × *A*^*failed*^ and *D* × *D*^*failed*^ are *AD* × *AD* generative models that were designed to violate the assumptions of the *A* × *A* and *D* × *D* models, respectively (see further Section Materials and methods). The results in Fig 4 shows that, for data generated from these specific models, there is no universal winning test strategy. However, both the *G* × *G joint* and *AD* × *AD joint* are generally among the best test under each generative model. Surprisingly, and in contrast to a single variant association test [28], the *A* × *A* test generally has a large loss of power when the generative model underlying the data is not *A* × *A*. Separate testing of parameters in the *G* × *G separate* or *R*/*D* × *R*/*D* tests sometimes incur a small loss of power compared to the joint tests. This can be expected because the application of Bonferroni correction in the separate test implicitly assumes independence of the individual interaction parameters, while the joint test accounts for any correlation among them; this will, overall, result in a small power advantage for the joint test.

**Fig 5:**
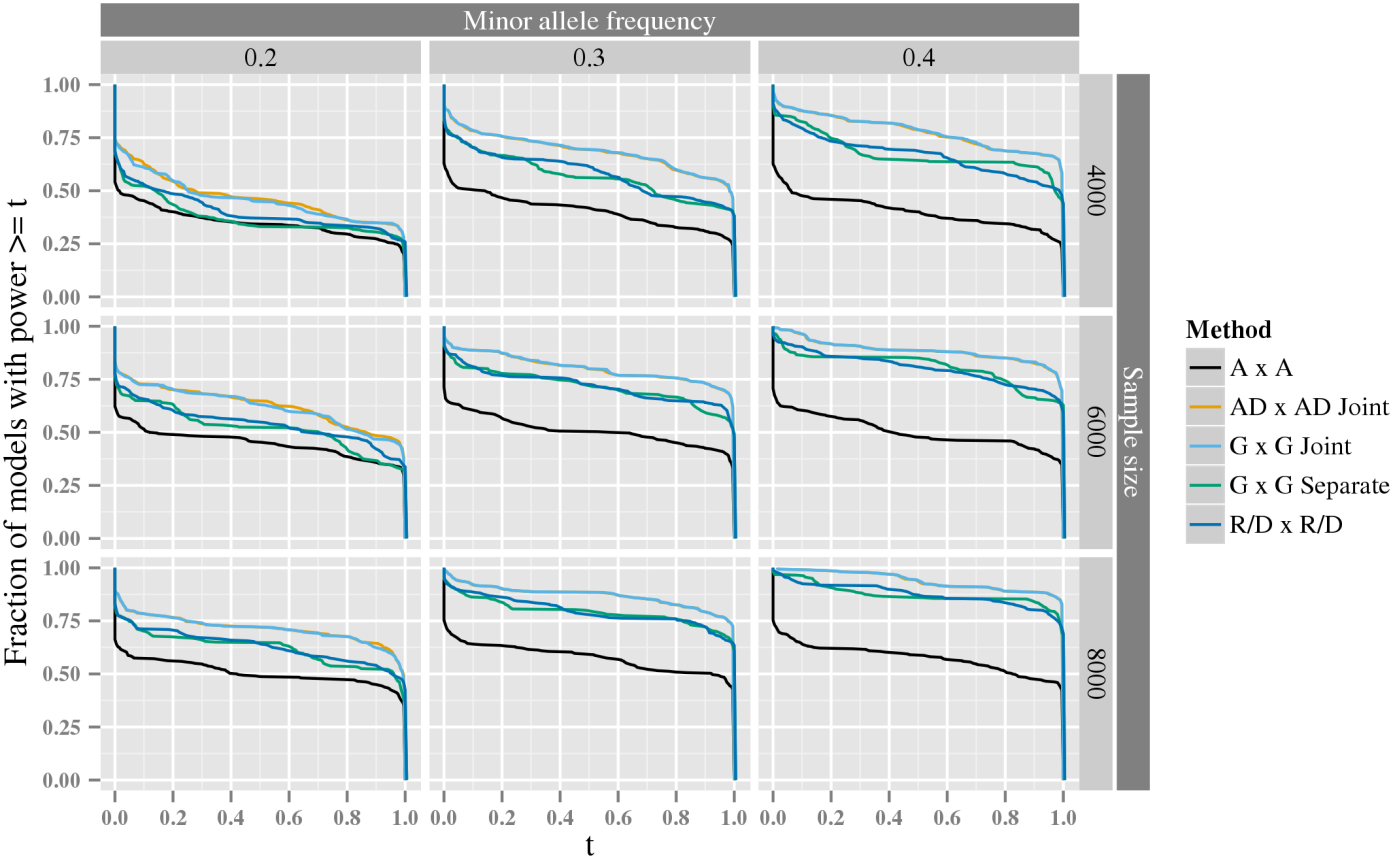
The exceedence distribution of power over all possible interaction generative models with a specific heritability. For each plot, the *x*-axis shows a threshold, *t*, for power to detect an interaction among 10^12^ variant pairs, and the corresponding y-axis shows the fraction of generative models, for which the analysis have a power greater than or equal to *t*. The rows correspond to the sample size. The columns correspond to the minor allele frequency of both variants in the pair. The line for the *AD* × *AD joint* test is often obscured by the line for the *G* × *G joint* test.

In the second experiment, we investigate the statistical power for the same tests over a large number of randomly sampled generative models. The results in Fig 5 reinforce those from the first power experiment. The joint tests, *G* × *G joint* and *AD* × *AD joint*, consistently perform best, followed by the *G* × *G separate* and *R*/*D* × *R*/*D* tests (where each parameter is tested separately), whereas the *A* × *A* test has on average 20% lower power. There is, as expected, no difference between the *G* × *G joint* and *AD* × *AD joint* tests. Despite the increased number of parameters of the joint tests these results holds also for lower sample sizes, see S3 Fig. However, we can not exclude that when many additional covariates are required, the tests with fewer parameter might gain statistical power.

### Biological data analysis

We applied the *G* × *G joint* test in two different association analyses aimed at the quantitative outcome blood concentration of Lp(a) lipo-protein particles [29] and a case-control outcome for myocardial infarction (MI), respectively. Our results, above, show that joint tests using saturated models has the best power. Moreover, joint tests using the *G* × *G* and *AD* × *AD* parameterizations are equivalent; our choice to use the *G* × *G* is arbitrary, but perhaps it is more easy to directly relate to the actual genotypes.

The link family test was used to test for link function invariance. As this test is quite computer-intensive, we apply the following strategy for each association analysis: We first perform the large-scale discovery analysis using the *canonical* link function in the respective analysis. The significant interactions from the discovery are then re-analyzed including the full test for link function invariance. The full link function invariance test is also applied in the replication analysis.

#### Lipoprotein(a)

We performed a classic discovery-replication analysis using quantitative data for circulating Lp(a). Lp(a) is an LDL-like lipoprotein particle also containing apolipoprotein [30], circulating in the blood. The concentration of circulating Lp(a) has been associated to coronary heart disease [31] and has attracted attention as a possible target for lipid-lowering therapies [32]. For the discovery we used the PROCARDIS cohort [33], comprising in total 8,112 individuals genotyped for 566,865 variants, while replication was done in the SCARF-SHEEP cohort [34, 35], comprising 2,345 individuals and 116,540 variants.

A full chip-wide analysis, testing all possible pairs from *L*, the set of variants in approximate linkage equilibrium (≈10^10^ pairs), resulted in no variant pair passing the very severe Bonferroni-corrected significance level (*p* < 10^−12^).

We therefore investigated a variance heterogeneity-based genome-wide association study (vGWAS) [36] approach as an *a priori* selection procedure for single variant interaction candidates The vGWAS analysis is claimed to be independent of, and not biasing ensuing interaction analysis; in fact a theoretical proof is provided [36]. In a vGWAS, each variant is first tested with a heterogeneity test (in this case the Brown-Forsythe test [37]). Variants that display sufficient variance heterogeneity comprise the set *V*. We used a liberal p-value threshold of 10^−4^. The final interaction analysis was performed on all pairs between *V* and *L*. A single variant pair (rs13209686-rs1884480) was significant from this analysis (adjusted *p* = 0.0306; Table 2).

**Table 2:** Association results for the discovery and fine-mapping analyses.

Analysis	SNP 1	SNP 2	df	N	*P*_*can*_	Adjusted *P*_*can*_	*P*_*linkinv*_	λ	Closest gene 1	Closest gene 2
Discovery	rs13209686	rs1884480	1	3677	2.42 ⋅ 10^−09^	0.0306	2.05 ⋅ 10^−07^	2	*SLC22A1*	*AGPAT4*
Fine-map	rs3103353	rs9458157	3	3688	1.26 ⋅ 10^−09^	NA	5.96 ⋅ 10^−09^	0	*SLC22A2*	*AGPAT4*
Fine-map	rs3103352	rs9458157	3	3673	1.05 ⋅ 10^−09^	NA	4.85 ⋅ 10^−09^	0	*SLC22A2*	*AGPAT4*

Because the variant pairs in the first analysis were first pruned for LD, we fine-mapped the association signal. For each of the variants *i* ∈ (1, 2) of the significant variant pair from the discovery analysis, we defined the set *F*_*i*_ of all variants in close proximity of *i*, and then analyzed all pairs of variants from *F*_1_ and *F*_2_. The top three pairs in the region are shown in Table 2. Only one of these variant pairs, comprising rs3103353 and rs9458157, was available for replication in our replication cohort SCARF-SHEEP. The replication result (Table 3) suggests that the discovery signal indeed replicates (*p* = 4.7378*e* − 07); the value of λ is consistent between discovery and replication and indicates that link should increase faster than linearly. Lastly, we performed a fixed-effect meta-analysis of the PROCARDIS and SCARF-SHEEP results for the rs3103353-rs9458157 pair. This resulted in a substantially stronger signal (*p* = 1.06 ⋅ 10^−12^; Table 3), indicating consistent effects in the two cohorts. This is also strengthened by the consistency of the estimated models for the two cohorts (S2 Fig), showing markedly lower expected Lp(a) levels for the double minor homozygote.

**Table 3:** Replication analysis of LP(a).

Cohort	SNP 1	SNP 2	N	P	λ
PROCARDIS	rs3103353	rs9458157	3688	5.96 ⋅ 10^−09^	0
SCARF-SHEEP	rs3103353	rs9458157	2343	6.97 ⋅ 10^−07^	0
Combined	rs3103353	rs9458157	6031	1.06 ⋅ 10^−12^	NA

Both rs3103353 and rs9458157 are located in the proximity of the previously associated LPA locus [29], but are not in LD (*r*^2^ = 0.0219008 and *D*′ = 0.186003). Functional annotation analysis using HaploReg [38](S3 Table) showed that there is previous evidence that rs3103352 is an eQTL for *SLC22A2* [39], while rs9458157 is situated in an intron of *AGPAT4*. Both variants are situated in regions with histone or methylation patterns associated with enhancer activity—in adipose tissue and in blood or cardiovascular tissue, respectively. Gene Ontology analysis [40](S4 Table) indicated that *SLC22A2* is a membrane transporter of organic cations, including histamine, choline and dopamine, while *AGPAT4* is involved in metabolic processes related to triglyceride and phospholipid metabolism.

Because of the proximity to the LPA locus, it is of interest to investigate the relation of this interaction to other single SNP associations in LPA, in particular those identified in previous studies (e.g., rs3798220 and rs10455872 [29] and rs41272114 [41]). We performed an additional analysis in the replication cohort, where we first identified all single variants associated to Lp(a), and then included each of these variants in turn as a covariate in the interaction analysis of the rs3103353-rs9458157 pair. The result (Supplementary table S10 Table) showed that the interaction association is not diminished by the additional covariate variants, suggesting that the identified interaction and neither of the associated single variants are proxies for the same association. However, the previously published associated variants rs10455872 and rs41272114 were not genotyped in our cohorts. Thus, we cannot test the possibility that the discovered interaction reflects the same association as one of these two variants or, for that matter, of any unsampled variant associated to Lp(a) in our cohort (i.e., either the interaction being a proxy for the single-variant [42] or vice versa [43]). We therefore performed a simulation study where we, using the PROCARDIS genotype data for chromosome 6, generated a continuous phenotype from a randomly picked single variant and then applied the same interaction analysis as for the biological data, but with different combinations of LD-pruning and vGWAS selection of variants; this was repeated 200 times (for details see S2 Text). The results (S8 Fig) shows that, while both approaches involving no pruning or LD-pruning controls the family-wise error rate (FWER), vGWAS does indeed inflate the FWER substantially. This contradicts the claim by [36] that vGWAS does not affect ensuing interaction analysis, this is likely caused by LD between tested variants which is assumed to be absent in the theoretical proof of Pare et al. [36]. To conclude, we cannot exclude the possibility of the existence of unsampled variants that display the same association as the discovered interaction between rs3103353 and rs9458157.

#### Myocardial infarction

MI is one of the common endpoints of coronary artery disease (CAD). Typically for complex diseases, such as CAD (and MI), very large sample sizes are needed for GWAS. Major collaborative efforts aimed at genetic analyses of CAD are therefore being performed, typically in large-scale meta-analyses driven by international consortia. As a proof-of-concept, we performed a two-cohort meta-analysis of gene-gene interactions associated to myocardial infarction (MI).

The first cohort was obtained by augmenting the PROCARDIS MI case-control data with control data from the WTCCC [44] (resulting in a total of 10,139 samples for analysis), the second cohort was the MIGEN cohort [45] (6,042 samples). This analysis only included variants that were genotyped in both cohorts (132,181 variants). We tested for interaction between all pairs among variants (≈ 8.74 ⋅ 10^9^ pairs) using a fixed effect meta-analysis, as described above in Section Fast estimation and testing of interaction in generalized linear models. No variant pair was declared significant using the Bonferroni correction (*p* < 5.72 ⋅ 10^−12^), the top five variant pairs (best p-value *p* = 1.91 ⋅ 10^−10^) are shown in S9 Table.

## Discussion

Our major contribution is computationally efficient tests for the complete family of GLMs, applicable to any combination of parameterization and link function. Our tests can be used for case-control studies as well as studies of quantitative traits. We have also shown how this methodology facilitates computationally efficient meta-analyses.

The *G* × *G* and *AD* × *AD* tests clearly had the overall best power to detect interaction. However, for certain data sets generated from specific interaction models, the *G* × *G separate* test and the *R*/*D* × *R*/*D* test performed comparable to the joint saturated full GLMs. Interestingly, the commonly used additive test had the overall worst power performance among the evaluated tests. It had strikingly poor power in several experiments. Nevertheless, for low minor allele frequencies the difference in power was less pronounced. In conclusion, our empirical results together with the equivalence of full saturated models, suggest that tests using saturated full GLMs are superior for interaction studies in general.

It is important that high power does not come at the expense of control of the false positive rate (FPR). Our simulations show that, under certain circumstances, incorrect parameterization in unsaturated or constrained GLMs may lead to incorrect interaction inferences. Specifically, when the data includes LD, tests using the constrained *R*/*D* × *RD* or the unsaturated *A* × *A* GLMs failed to control FPR, while it was still controlled by the tests using saturated full GLMs. Moreover, also for data generated without LD, but then only when generated with a Binomial dispersion distribution, incorrect parameterization caused the *A* × *A* test to display substantial FPR inflation.

We demonstrate the applicability of our method on experimental data aimed at Lp(a). Levels of circulating Lp(a) have an inverse association with coronary heart and are predominantly regulated by genetic variation in proximity of the LPA gene [29]. Although the mechanism remains elusive, it has been hypothesized that the association between Lp(a) levels and CVD is indeed causal, in part because a number of genetic polymorphisms have effects on both LP(a) levels and risk of cardiovascular events. We discovered a new genome-wide significant interaction between two variants in proximity to the LPA locus. This could potentially reflect an interaction between *AGPAT2*, involved in triglyceride metabolism, and *SLC22A2*, a transmembrane transporter, which previously has been indicated as involved in lipoprotein metabolism [46]. However, as often is the case, we cannot exclude the possibility that the discovered interaction may be a proxy for an unsampled, either single variant or another interaction, association.

We demonstrated the applicability of our meta-analysis method by analyzing two MI case-control cohorts. The genetic architecture of MI is known to be complex and single variant analysis has so far explained only a small fraction of its heritability. This motivated us to investigate the potential impact of interactions on MI. Our meta-analysis did not result in any interactions of genome-wide significance, which require passing the severe Bonferroni-corrected threshold *p* ≤ 5.72 ⋅ 10^−12^.

The link function, a.k.a. the “scale”, is a crucial component of GLMs that relates the linear predictors to the phenotype mean. Because the link function used in a test can have a considerable impact on the resulting FPR, it is of interest whether the interaction is invariant of the scale, and a few tests for this have been devised. We applied the link family test, which evaluates invariance across a family of link functions [22, 23]. This test is unfortunately computationally demanding and can be restrictive for large-scale analyses. To circumvent this, we used the so-called *canonical* link function in the large-scale discovery phase, and performed the test for link function invariance only on those interactions that passed the discovery phase.

Alvarez-Castro and Carlborg [18] derived conditions for when the parameterization is orthogonal in a linear model. Parameterization orthogonality facilitates decomposition of the variance into components corresponding to main and interaction effects. For a GLM the concept of orthogonality is complicated by the link function and the genotypic dependence of the variance (see S1 Text). Nevertheless, our tests are applicable to both orthogonal and non-orthogonal parameterizations alike.

Our test is currently limited to discrete genotype data, i.e., directly genotyped or hard-called imputed data [47]. Meta-analysis of cohorts with non-overlapping genotype data requires imputed data. However, with currently available algorithms for incorporating imputation uncertainty in interaction tests, this would substantially compromise computational efficiency. Similarly, in the present implementation it is not obvious how to include covariates in the analysis. A solution that we are currently working on could be to use the GLM weights to model both imputation uncertainty and covariate adjustment. Ideally, this will retain the computational efficiency at the cost of statistical efficiency. However, for the time being, the simplest solution to the covariate issue is a two-step strategy, which, *a posteriori* to the initial analysis, fits a standard GLM including relevant covariates for the identified interactions and checks that the results still hold (this approach was used in the Lp(a) replication analysis). If this causes too many pairs to be identified, an alternative that is commonly applied in univariate meta-analyses is to first regress out relevant covariates and then model the residuals. Because residuals are expected to be normally distributed for all GLM models this can be generally applied; however, some interpretation is lost.

## Materials and methods

### False positive rate

We first considered two scenarios that may produce false positives: submodels in which one or more assumptions fails to hold, and tests where the incorrect link function is used. In both experiments, we evaluated the following five different tests: the *G* × *G joint*, *AD* × *AD joint*, *G* × *G separate*, *A* × *A* and *R*/*D* × *R*/*D* tests.

In the first experiment we investigated FPR where the data violates two model assumptions, erroneous parameterization and presence of linkage disequilibrium (LD). LD was measured by Lewontin’s *D* (*LD*) and we used two cases, high linkage (*LD* = 0) and low linkage (*LD* = 0.8). The genotypes were generated according to the following probability distribution

$$\begin{array}{c} \left(\begin{array}{ccc} p_{00}^{2} & 2p_{00}p_{01} & p_{01}^{2}\\ 2p_{00}p_{10} & 2p_{00}p_{11}+2p_{01}p_{10} & 2p_{01}p_{11}\\ p_{10}^{2} & 2p_{10}p_{11} & p_{11}^{2} \end{array}\right) \end{array}$$ (4)

where

$$\begin{array}{c} \left(\begin{array}{cc} p_{00} & p_{01}\\ p_{10} & p_{11} \end{array}\right)=\left(\begin{array}{cc} (1-r)(1-q)+D & r(1-q)-D\\ (1-r)q-D & rq+D \end{array}\right) \end{array}$$ (5)

and *q* is the minor allele frequency of the first variant, and *r* is the minor allele frequency of the second variant. The parameter *D* = *LD* ⋅ *D*_*min*_ where *D*_*min*_ = min(1 − *qr*, 1 − (1 − *q*)(1 − *r*), *q*(1 − *r*), (1 − *q*)*r*). The minor allele frequency was set to *q* = *r* = 0.3 and the sample size to 4,000. For each genotype, continuous phenotypes were then generated using six different null GLMs, obtained as the combinations of (i) a linear predictor from either of the *A* × *A*, *R* × *A* and *R* × *D* parameterizations, (ii) the identity link function, and (iii) either a Binomial and normal dispersal distribution (parameters can be seen in S5 Table). For each parameter combination we generated 1,000 data sets and estimated the false positive rate as the average number of incorrectly identified pairs.

In the second experiment, we investigated the false positive rate when the link function is misspecified. Specifically, we generated the phenotype using a log link function, while the tests were performed using the identity link function. The genotypes were generated under Hardy-Weinberg equilibrium with a minor allele frequency of 0.3 for both variants. We used a GLM with the *A* × *A* linear predictor, the identity link function and a normal dispersion distribution to generate phenotype from each genotype; we varied the size of the second variant’s main effect between −0.4 and 0.4 and the sample size over 2000, 3000 and 4000 (parameters are shown in S6 Table). For each parameter combination, we generated 1000 data sets and estimated the false positive rate as described above.

### Statistical power

We performed two different simulation experiments for statistical power; one smaller comprising a set of specific GLMs and one larger comprising a wide range of interaction models. In both experiments we generated a continuous phenotype from a Normal dispersion distribution using the identity link function. We evaluated the same tests as in the FPR experiments, described above.

In the small power experiment we generated data from six different parameterizations, *A* × *A*, *A* × *A*^*failed*^, *R* × *D*, *D* × *D*, *H* × *H*, and *D* × *D*^*failed*^. The *A* × *A*^*failed*^ and *D* × *D*^*failed*^ are *AD* × *AD* models designed to violate the assumptions of the *A* × *A* and *D* × *D*, respectively, that is, in the *A* × *A*^*failed*^, the values of the interaction parameters are switched with respect to those in a *A* × *A*, while in the *D* × *D*^*failed*^, half of the interaction parameters are in the opposite and wrong direction compared to *D* × *D*. We generated genotypes for 4000 individuals under Hardy-Weinberg equilibrium with a minor allele frequency 0.3 for both variants. For each genotype, we then generated phenotypes from each GLM. We varied the effect size between -1.0 to 1.0; depending on the selected parameterization, this required modification either of a single or multiple parameters in ***β*** (parameters can be seen in S7 Table). For each effect size, we generated 1000 data sets and estimated the statistical power of each test as the average number of correctly identified pairs under Bonferroni correction assuming 10^12^ variant pairs.

In the large power experiment, we focused on different *G* × *G* parameterizations from a range of parameter combinations that can be found in S8 Table—notice that, since this is a saturated full parameterization, this approach will cover a large number of other, unsaturated and saturated, constrained and full, parameterizations. The genotypes were generated under Hardy-Weinberg equilibrium and we varied the allele frequency between 0.2, 0.3 and 0.4 (for both variants), and the sample size between 2000, 3000 and 4000. For each genotype a phenotype was generated using a linear predictor from the *G* × *G* parameterizations described above, the identity link function and the Normal dispersion distribution. For each parameter combination we generated 1000 data sets and estimated the statistical power as described above (using a Bonferroni correction assuming 10^12^ variant pairs).

### Ethics statement

The PROCARDIS study was approved by the Regional Ethics Review Board at Karolinska Institutet, Stockholm in Sweden (approval number 98-482 and 03-491) and by the Institutional Review Boards of the Mario Negri Institute, Milano in Italy, the University of Munster, Munster, in Germany, and the University of Oxford, Oxford, United Kingdom. The PROCARDIS study was supplemented with controls from the WTCCC study, UK. The WTCCC was approved by the relevant research ethics committees. All study participants provided their written informed consent to participate in the study, which was conducted in accordance with the Helsinki Declaration.

The SCARF and SHEEP studies were approved by the local ethics committees at Karolinska Hospital, Stockholm (approval number 95-397 and 02-091), and Karolinska Insitutet, Stockholm (approval number 01-097), respectively.

The MIGEN data was accessed from dbGAP (access.nr. phs000294.v1.p1) [45]; all participants in the MIGEN studies gave written informed consent in accordance with the guidelines of local ethical committees.

### Biological data

The PROCARDIS multicentre study was designed to investigate early onset CAD. Cases with a documented CAD event before the age of 66 years were collected from Sweden, the UK, Germany and Italy. The full PROCARDIS cohort comprise 8410 cases and 5188 matched controls free from CAD. Here we have used a subset of the PROCARDIS cohort previously genotyped with the Illumina Human1M Quad chip and the Illumina Human610K chip [33]. We only included unrelated individuals genotyped on these chips. The intersection of these chips contain 566,865 variants. The subset of PROCARDIS used in this study depends on the phenotype. Lp(a) plasma levels were measured in 3,741 individuals. For the meta-analysis aimed at MI, the PROCARDIS cohort was extended with 5,667 control samples from the Wellcome Trust Consortium (WTCCC) [44]. This resulted in a total of 2,809 cases and 7,330 controls for the MI disease phenotype.

The SCARF [35] and SHEEP [34] cohorts included unrelated MI cases from the Stockholm region of Sweden, with age and sex-matched controls collected from the general population of the same region. The comparable design and demographics of the 2 cohorts means that they can be combined as one cohort. The SCARF-SHEEP cohort was previously genotyped with the CardioMetabo chip, a custom Illumina iSelect genotyping array that targets genetic variants likely to be involved in metabolic and cardiovascular disorders [48]. The chip contains 116,540 variants. Lp(a) levels were measured in 2,345 individuals.

The MIGEN cohort is a case-control study aimed at investigating the genetic basis of MI [45]. The cohort contains samples from 6 collection sites: Boston, MA (Masschusetts General Hospital Premature Coronary Artery Disease Study), Seattle, WA (Heart Attack Risk in Puget Sound), Helsinki, Finland (FINRISK), Malmö, Sweden (Malmö Diet and Cancer Study), Barcelona, Spain (REGICOR), and Milan, Italy (Italian Atherosclerosis Thrombosis and Vascular Biology Working Group). The MIGEN cohort was previously genotyped with the Affymetrix Genome-wide Human SNP Array 6.0. The data was approved by and downloaded from dbGAP with accession *phs000294.v1.p1*. This data contains 3,068 cases and 2,957 controls.

### QC and analysis of biological data

We performed multiple experiments on biological data: exhaustive interaction scan in Lp(a), vGWAS scan in Lp(a), and a exhaustive meta analysis of CAD. All interaction tests was performed using the *G* × *G joint* test. In the discovery phase, a canonical link function and the appropriate dispersion distribution was used in an initial analysis. The significant pairs was then tested using the same *G* × *G joint* test, but using the link family test to step through a family of link functions; the latter approach was also used in the replication phase. For the continuous Lp(a) phenotype, the Normal dispersion distribution was used first with the canonical identity link and then with the symmetric Box-Cox family. For the binary MI case-control data, the Binomial dispersion distribution was used first with the logit link and then with Pregibon link family.

The Lp(a) phenotype was log-transformed and outliers were removed in both PROCARDIS and SCARF-SHEEP. For the exhaustive interaction scan we applied the following QC filters: minor allele frequency 0.05, genotyping rate 0.1. We then removed variants in linkage disequilibrium using the Plink “–indep-pairwise” option, using a window size of 100, step size of 5 and a LD threshold of *r*^2^ < 0.5 (we additionally check *D*′ for any significant interaction discoveries). This resulted in 180,947 variants, and we tested for interaction between all possible pairs of these.

For the vGWAS scan, we applied the same QC filters as above; the resulting set of SNPs is called *L*. We then tested for variance heterogeneity of each variant with a Brown-Forsythe test [37]. The set of variants with p-value less than 10^−4^ is called *V*. We constructed all pairs of one variant from *V* and one variant from *L* (1.26 ⋅ 10^7^ pairs) and tested these for interaction.

In the meta-analysis, we only considered variants that was genotyped both in MIGEN and PROCARDIS, this resulted in 132,181 variants. We applied the following QC filters: minor allele frequency > 0.05, genotyping rate > 0.1. We only tested pairs for which all genotypes had at least five samples in both cases and controls (8.17 ⋅ 10^9^ pairs).

For each analyzed cohort and phenotype combination we checked for potential p-value inflation (genomic inflation) due to population stratification. In all cases genomic inflation was low (see S4, S5, S6 and S7 Figs). The QQ-plots for the two Lp(a) cohorts (S6 and S7 Figs) showed a surprisingly large deviatin from the diagonal line, expected under the null mode. After investigation, it became clear that this inflation was due to substantial LD at the previously identified strongly associated Lp(a) locus on chromosome 6 [29].

### Software availability

A c++ implementation of the software can be accessed at https://github.com/mfranberg/besiq.

## Supporting information

S1 Text This text contains mathematical proofs of statements made in the main text. The text contains four sections: derivation of closed-form estimation of saturated full parameterizations, proof of that the fast estimation of unsaturated models is consistent, description of orthogonality for GLMs, and derivation of the fixed effect meta-analysis for Wald statistics.
(PDF)

S2 Text This text describes how the supporting experiments were set up, i.e., the impact of potentially associated sampled and unsampled variants on discovered associations of interaction pairs. (PDF)

S1 Fig Speed comparison between the likelihood ratio (LR) test and the closed-form Wald test (Wald). The left and right subplots are for binary and continuous phenotypes respectively. The x-axis is the total sample size, for the binary phenotype this value is cases plus controls. The y-axis is the average time required to compute the p-value per variant pair on a log-scale. The colors represent the algorithm used to compute the test statistics, “Plink” refer to the –epistasis-test in the Plink 1.90 Beta software, “Wald” refer to the Wald test, and “IRLS” refer to the likelihood ratio test implemented using the iteratively reweighed least squares algorithm.
(TIFF)

S2 Fig Comparison between the interaction model estimated in the discovery (left) and replication cohort (right). The x-axis is the number of minor alleles of the first variant. The y-axis is the expected value of the phenotype. The colors correspond to the number of minor alleles of the second variant.
(TIFF)

S3 Fig The exceedence distribution of power over all possible interaction generative models with a specific heritability—Smaller sample sizes. For each plot, the *x*-axis shows a threshold, *t*, for power to detect an interaction among 10^12^ variant pairs, and the corresponding y-axis shows the fraction of generative models, for which the analysis have a power greater than or equal to *t*. The rows correspond to the sample size. The columns correspond to the minor allele frequency of both variants in the pair. The line for the *AD* × *AD joint* test is often obscured by the line for the *G* × *G joint* test.
(TIFF)

S4 Fig Single variant QQ-plot for MI in PROCARDIS. The x-axis is the expected log p-values and the y-axis is the observed log p-values. There is little deviation from the diagonal line and genomic inflation is low (λ = 1.102).
(TIFF)

S5 Fig Single variant QQ-plot for MI in MIGEN. The x-axis is the expected log p-values and the y-axis is the observed log p-values. There is little deviation from the diagonal line and genomic inflation is low (λ = 1.100).
(TIFF)

S6 Fig Single variant QQ-plot for LP(a) in PROCARDIS. The x-axis is the expected log p-values and the y-axis is the observed log p-values. There is substantial deviation from the diagonal line; however, genomic inflation is low (λ = 1.109).
(TIFF)

S7 Fig Single variant QQ-plot for LP(a) in SCARF-SHEEP. The x-axis is the expected log p-values and the y-axis is the observed log p-values. There is substantial deviation from the diagonal line; however, genomic inflation is low (λ = 1.047).
(TIFF)

S8 Fig Family-wise error rates for different filtering strategies. The x-axis is the total heritability of the generated variants. The y-axis is the estimated family-wise error rate. The colors correspond to the number of causal variants. The dashed line is the 0.05 FWER threshold. The error bar of each point is the 95% confidence interval of the corresponding FWER estimate. Each subplot corresponds to 4 different pruning strategies: no pruning, only ld-pruning, only vGWAS pruning, and both ld-pruning and vGWAS pruning.
(TIFF)

S1 Table Misspecification bias for three common link functions. The first column is the name of the link function. The second column is the form of the link function. The third column is the bias when the link function is misspecified by a small perturbation ***ϵ*** using parameterization matrix *P*. Here diag(*x*) denotes the diagonal matrix with the vector *x* as the diagonal.
(PDF)

S2 Table Description of common dispersion distributions used for different phenotype types. The first column is the type of phenotype. The second column is the name of the dispersal distribution commonly used for the corresponding phenotype type. The third column is the canonical link function.
(PDF)

S3 Table Summary of HaploReg analysis for the LP(a) and MI interaction association results. The abbreviation ‘Enh’ stands for Enhancer and ‘Pro’ for Promoter, see the Roadmap epigenomics project for further details.
(PDF)

S4 Table Summary of gene ontology analysis for the LP(a) and MI interaction association results. Gene symbols for the closest genes (Tables 2 and 4) were used as search terms on the gene ontology website. Searches were limited to GO terms relating to Homo Sapiens and biological processes.
(XLSX)

S5 Table Parameters that underlie the first FPR experiment. The first column is the model, the second the LD of the model, the rest of the columns are the model parameters used in the simulation (described in the context of a saturated GLM; *σ* is the variance of the Normal dispersion distribution).
(PDF)

S6 Table Parameters that underlie the second FPR experiment. The first column is the effect size used on the x-axis in the plot. The rest of the columns are the normal model parameters (described in the context of a saturated GLM; *σ* is the variance of the Normal dispersion distribution).
(PDF)

S7 Table Parameters that underlie the small power experiment. The first column is the model, the second column is the effect size used on the x-axis of the corresponding plot, the rest of the columns are the parameters used in the simulation (described in the context of a saturated GLM; *σ* is the variance of the Normal dispersion distribution).
(PDF)

S8 Table Parameters that underlie the large power experiment. The first column is the variance of the normal distribution, the second column the intercept, column four to eight are the main effects. The last column denotes the sum of the interaction effect sizes, these were used to generate all possible interaction models where the mean of the non-zero effect sizes equaled this number.
(PDF)

S9 Table Results for the meta-analysis on myocardial infarction. For each variant pair, the p-value and sample size are given for the meta-analysis and the analyses of individual the MIGEN and the PROCARDIS cohorts.
(PDF)

S10 Table Results for the interaction test between rs3103353 and rs9458157 in SCARF-SHEEP when adjusting for genome-wide significant variant. For each genome-wide significant (*p* < 5 ⋅ 10^−8^) variant *v*, this table shows the likelihood ratio (LR), the p-value (p) and the sample size (N) for the test of association of the rs3103353-rs9458157 interaction pair, when adjusting for *v*.
(XLSX)
