# Supplementary material for: Fast and general tests of genetic interaction for genome-wide association studies
Source: PLoS Comput Biol. 2017 Jun 6;13(6):e1005556. doi: 10.1371/journal.pcbi.1005556 (PMC5478145; doi:10.1371/journal.pcbi.1005556)
Supplement: S1 Text — The text contains four sections: derivation of closed-form estimation of saturated full parameterizations, proof of that the fast estimation of unsaturated models is consistent, description of orthogonality for GLMs, and derivation of the fixed effect meta-analysis for Wald statistics. (PDF) [file pcbi.1005556.s001.pdf]

Supporting information S1 Text
for
Modeling genetic interactions using generalized linear models

Mattias Frånberg, Rona J Strawbridge, Anders Hamsten, Jens Lagergren, Bengt Sennblad

November 28, 2016

1 Generalized linear models for genetic data

1.1 Introduction to GLMs

A generalized linear model describes the distribution of random variable conditioned on one or more other random variables. The generalized linear model consists of two components: a distribution $f(y)$ in the exponential family and a link function g that maps the linear predictor to the mean parameter of the distribution. Let y_i for individual $i \in \{1...n\}$ be the random variable that we want to model conditioned on a set of covariates x_{ij} for $j \in \{1...m\}$. Let X denote the matrix of covariates, and $\boldsymbol{x_i}$ the vector of covariates corresponding to individual i. Vectors are denoted by bold face.

The expected value of the outcome $\mu_i = E[Y_i]$ for individual i is described as a function of the covariates

$$g(\mu_i) = \boldsymbol{x_i}\boldsymbol{\beta} = \eta_i,$$

where we introduce η_i to simplify notation later. Because the distribution belongs to the exponential family, the likelihood of the model can be written on the following form

$$f(y_i \mid x_i; \theta_i) = e^{\frac{y_i\theta_i - c(\theta_i)}{\phi}} h(y_i, \phi)$$

where θ_i is called the canonical parameter and has a one-to-one mapping to μ_i such that $\theta_i = \theta(\mu_i) = \theta(g^{-1}(\eta_i))$; ϕ is called the dispersion parameter and for a normal distribution with standard deviation σ we have $\phi = \sigma^2$; $h(y_i, \phi)$ is specific function for each distribution that only depends on y_i and ϕ; and finally $c(\theta_i)$ is a normalization constant that normalizes the distribution to 1. The normalization constant has the following important properties

$$\begin{aligned} -\frac{\partial c(\theta_i)}{\partial \theta_i} &= E[Y_i \mid \boldsymbol{x_i}] = \mu_i \\ -\frac{\partial^2 c(\theta_i)}{\partial \theta_i^2} &= \frac{\partial \mu_i}{\partial \theta_i} = Var[Y_i \mid \boldsymbol{x_i}, \phi = 1] = v(\mu_i) \end{aligned}$$

The likelihood for a sample of size n is

$$f(\boldsymbol{y} \mid X; \boldsymbol{\theta}) = \prod_{i=1}^{n} e^{\frac{y_i\theta_i - c(\theta_i)}{\phi}} h(y_i, \phi)$$

and the log-likelihood

$$\log f(\boldsymbol{y} \mid X; \boldsymbol{\theta}) = \sum_{i=1}^{n} \frac{y_i\theta_i - c(\theta_i)}{\phi} + \log h(y_i, \phi)$$

The score function of a parameter is the partial derivative of the log-likelihood with respect to that parameter. This is used to find maximum likelihood estimates of the parameters by solving $U(\boldsymbol{\beta}) = 0$. The score function for β_j in a generalized linear model is

$$\begin{aligned} U(\beta_j) &= \frac{\partial \log f(y \mid X; \theta)}{\partial \beta_j} = \sum_{i=1}^{n} \frac{y_i - c'(\theta_i)}{\phi} \frac{\partial \theta_i}{\partial \mu_i} \frac{\partial \mu_i}{\partial \eta_i} \frac{\partial \eta_i}{\partial \beta_j} \\ &= \sum_{i=1}^{n} \frac{y_i - \mu_i}{\phi} \frac{1}{v(\mu_i)} \frac{1}{g'(\mu_i)} x_{ij} \end{aligned}$$

where $v(\mu_i) = Var[Y_i \mid \boldsymbol{x_i}, \phi = 1]$ and g' is the derivative of the link function.

The Fisher information matrix is used to obtain the covariance matrix between a set of estimated parameters, that in turn is used for hypothesis testing. In a generalized linear model the Fisher information between two parameters β_j and β_k is derived by

$$\begin{aligned} \mathcal{I}(\beta_j, \beta_k) &= -E\left[\frac{\partial}{\partial \beta_k} U(\beta_j)\right] = -E\left[\frac{\partial}{\partial \beta_k} \sum_{i=1}^{n} \frac{y_i - \mu_i}{\phi} \frac{x_{ij}}{v(\mu_i) g'(\mu_i)}\right] \\ &= -E\left[\sum_{i=1}^{n} -\frac{1}{\phi} \frac{x_{ij}}{v(\mu_i) g'(\mu_i)} \frac{\partial \mu_i}{\partial \eta_i} \frac{\partial \eta_i}{\partial \beta_k} + \frac{y_i - \mu_i}{\phi} \frac{\partial}{\partial \beta_k} \frac{x_{ij}}{v(\mu_i) g'(\mu_i)}\right] \\ &= \sum_{i=1}^{n} \frac{x_{ij}}{\phi v(\mu_i) g'(\mu_i)} \frac{\partial \mu_i}{\partial \eta_i} \frac{\partial \eta_i}{\partial \beta_k} = \sum_{i=1}^{n} \frac{x_{ij} x_{ik}}{\phi v(\mu_i) g'(\mu_i)^2} \end{aligned}$$

1.2 The score and Fisher Information in genetic models

Now we can start to put in assumptions about the parameterization. Because we are working with discrete genotypes both the score and Fisher information can be further simplified. Let there be H possible genotypes and $h_i \in \{1...H\}$ denote the genotype of individual i, let $P = \{p_{hk}\}$ be a matrix where row h corresponds to genotype h, and p_{hk} is the value of covariate k for genotype h (the value multiplied by β_k). Furthermore let μ_h, compared to μ_i, be the mean of the phenotype for all individuals with genotype h. Now because there are a finite number of genotypes there will be repeated rows in X, we will condense this by using P along with counts for each genotype n_h and a corresponding sufficient statistic t_h for the mean μ_h. The score function is simplified as follows

$$\begin{aligned} U(\beta_j) &= \sum_{i=1}^{n} \frac{y_i - \mu_i}{\phi} \frac{x_{ij}}{v(\mu_i) g'(\mu_i)} = \sum_{i=1}^{n} \frac{y_i - \mu_{h_i}}{\phi} \frac{p_{h_i j}}{v(\mu_{h_i}) g'(\mu_{h_i})} \\ &= \sum_{h=1}^{H} \frac{t_h - n_h \mu_h}{\phi} \frac{p_{hj}}{v(\mu_h) g'(\mu_h)} = \sum_{h=1}^{|H|} \frac{t_h - n_h \mu_h}{\phi} \frac{p_{hj}}{v(\mu_h) g'(\mu_h)} \end{aligned}$$

where $t_h = \sum_{i|h_i=h} y_i$. In matrix notation this is written

$$U(\boldsymbol{\beta}) = PW\left(\frac{\boldsymbol{t} - N\boldsymbol{\mu}}{\phi}\right)$$

where W is a diagonal matrix containing $w_{hh} = \frac{1}{v(\mu_h) g'(\mu_h)}$, and N a diagonal matrix containing $n_{hh} = n_h$. The maximum likelihood estimates are found by solving $U(\boldsymbol{\beta}) = 0$ i.e.

$$U(\boldsymbol{\beta}) = 0 \Leftrightarrow PW\boldsymbol{t} = PWN\boldsymbol{\mu}$$

Assuming that the number of parameters equals the number of genotypes (a saturated model), we can simplify this to

$$\boldsymbol{t} = N\boldsymbol{\mu} \Leftrightarrow \boldsymbol{\mu} = N^{-1}\boldsymbol{t} \Leftrightarrow g^{-1}(P\boldsymbol{\beta}) = N^{-1}\boldsymbol{t} \Leftrightarrow \boldsymbol{\beta} = P^{-1}g(N^{-1}\boldsymbol{t})$$

That is, we can directly solve for $\boldsymbol{\beta}$ without iteration. Furthermore, the Fisher information can also be simplified

$$\mathcal{I}(\beta_j, \beta_k) = \sum_{i=1}^{n} \frac{x_{ij}x_{ik}}{\phi v(\mu_i)g'(\mu_i)^2} = \sum_{i=1}^{n} \frac{p_{h_i j}p_{h_i k}}{\phi v(\mu_{h_i})g'(\mu_{h_i})^2} = \sum_{h=1}^{H} \frac{n_h p_{hj} p_{hk}}{\phi v(\mu_h)g'(\mu_h)^2}$$

where n_h is the number of individuals with genotype h. The Fisher information matrix has a simple form and can be written

$$\mathcal{I} = P'VP$$

where V is a diagonal matrix containing the terms $v_{hh} = \frac{n_h}{\phi v(\mu_h)g'(\mu_h)^2}$, and $'$ denotes the transpose. More importantly the inverse of the Fisher information, i.e. the covariance matrix between the parameters, has a rather simple form and can be written

$$C = \mathcal{I}^{-1} = P^{-1}W^{-1}P'^{-1}$$

1.3 The variance-covariance matrix for $G \times G$

In the genotypic parameterization the design matrix is

$$P = \begin{pmatrix} 1 & 0 & 0 & 0 & 0 & 0 & 0 & 0 & 0 \\ 1 & 1 & 0 & 0 & 0 & 0 & 0 & 0 & 0 \\ 1 & 0 & 1 & 0 & 0 & 0 & 0 & 0 & 0 \\ 1 & 0 & 0 & 1 & 0 & 0 & 0 & 0 & 0 \\ 1 & 1 & 0 & 1 & 0 & 1 & 0 & 0 & 0 \\ 1 & 0 & 1 & 1 & 0 & 0 & 1 & 0 & 0 \\ 1 & 0 & 0 & 0 & 1 & 0 & 0 & 0 & 0 \\ 1 & 1 & 0 & 0 & 1 & 0 & 0 & 1 & 0 \\ 1 & 0 & 1 & 0 & 1 & 0 & 0 & 0 & 1 \end{pmatrix}$$

the covariance matrix is in this case derived to

$$\begin{matrix} c_1 + c_2 + c_4 + c_5 & c_1 + c_4 & c_1 + c_2 & c_1 \\ c_1 + c_4 & c_1 + c_3 + c_4 + c_6 & c_1 & c_1 + c_3 \\ c_1 + c_2 & c_1 & c_1 + c_2 + c_7 + c_8 & c_1 + c_7 \\ c_1 & c_1 + c_3 & c_1 + c_7 & c_1 + c_3 + c_7 + c_9 \end{matrix}$$

where $c_k = \frac{\phi v(\mu_k)g'(\mu_k)^2}{n_k}$.

2 Fast estimation of unsaturated models is consistent

We want to estimate $\hat{\boldsymbol{\beta}}$ of a unsaturated model. We first saturate the model to get $\hat{\boldsymbol{\beta}}_S$. We have the following relationship between the parameters of the saturated and unsaturated model $\boldsymbol{\beta}_S = S\boldsymbol{\beta}$. Given an estimate of the covariance matrix C_S of the saturated parameters we can estimate $\boldsymbol{\beta}$ and their covariance matrix C by

$$\hat{\boldsymbol{\beta}} = (S^T\hat{C}_S^{-1}S)^{-1}S^T\hat{C}_S^{-1}\hat{\boldsymbol{\beta}}_S$$

and

$$\hat{C} = S^T\hat{C}_S^{-1}S$$

Theorem 1. *If $\hat{\boldsymbol{\beta}}_S$ is estimated by maximum likelihood in a model satisfying the standard regularity conditions for maximum likelihood, then the estimator $\hat{\boldsymbol{\beta}} = (S^T\hat{C}_S^{-1}S)^{-1}S^T\hat{C}_S^{-1}\hat{\boldsymbol{\beta}}_S$ is consistent i.e.*

$$(S^T\hat{C}_S^{-1}S)^{-1}S^T\hat{C}_S^{-1}\hat{\boldsymbol{\beta}}_S \xrightarrow{p} (S^TC_S^{-1}S)^{-1}S^TC_S^{-1}S\boldsymbol{\beta} = \boldsymbol{\beta}$$

Proof. We will here utilize the continuous mapping theorem (CMT [1]). Firstly, by maximum likelihood theory we have that $\hat{\boldsymbol{\beta}}_S \xrightarrow{p} \boldsymbol{\beta}_S = S\boldsymbol{\beta}$, secondly we then have that $\hat{C}_S = I(\hat{\boldsymbol{\beta}}_S)^{-1} \xrightarrow{p} I(\boldsymbol{\beta}_S)^{-1} = C_S$ by CMT. Using Slutsky's theorem we can therefore conclude that $(\hat{\boldsymbol{\beta}}_S, \hat{C}_S) \xrightarrow{p} (\boldsymbol{\beta}_S, C_S)$ (notice, for random variables converging to fixed numbers $\xrightarrow{p}$ and $\xrightarrow{d}$ are equivalent). We can now apply CMT using the joint variables on the full expression to find

$$(S^T\hat{C}_S^{-1}S)^{-1}S^T\hat{C}_S^{-1}\hat{\boldsymbol{\beta}}_S \xrightarrow{p} (S^TC_S^{-1}S)^{-1}S^TC_S^{-1}S\boldsymbol{\beta} = \boldsymbol{\beta}$$

thus the estimator is consistent. □

3 Orthogonal generalized linear models

In a genetic model it is common to measure the contribution of a specific parameter in terms of the additional variance that it explains. For a linear model, i.e. $g(x) = x$ and $Var(Y \mid p_h) = \sigma^2$, this contribution can be found by looking at the total variance of Y

$$Var(Y) = Var(\boldsymbol{\beta}x + \epsilon) = Var(\boldsymbol{\beta}x) + Var(\epsilon)$$

. In general this contribution does not have a simple expression, but if the paramterization is *orthogonal*, i.e. $Cov(x_i, x_j) = 0$, then it can be calculated by

$$Var(Y) = \sum_{i=1}^{H} b_i^2 Var(x_i) + \sigma^2$$

and the fraction of variance explained by b_i is therefore $b_i^2 Var(x_i)/Var(Y)$. This concept can be generalized to a GLM by considering the deviance instead of the variance. The deviance explained is defined

$$D_e = \frac{-2(l(y;\hat{\beta}_0) - l(y;\hat{\boldsymbol{\beta}}))}{-2(l(y;\hat{\beta}_0) - l(y;y))}$$

where $l(y, \hat{\boldsymbol{\beta}})$ denotes the likelihood of y in the maximum likelihood point $\boldsymbol{\beta}$, $l(y, \beta_0)$ denotes the likelihood of y when only using an intercept $\hat{\beta}_0$, and $l(y; y)$ denotes the likelihood when there is one parameter per sample, i.e. the maximum obtainable likelihood. The deviance, with respect to orthogonality, is cumbersome to work directly with, we therefore use the large sample approximation

$$D_e \approx \frac{\boldsymbol{\beta}'((\mathcal{I}^{-1})_{-\beta_0})^{-1}\boldsymbol{\beta}}{-2(l(y;\hat{\beta}_0) - l(y;y))}$$

This means, that for the deviance to separate into a sum in which each term contains a single β_i, the matrix $((\mathcal{I}^{-1})_{-\beta_0})^{-1}$ must be diagonal. This matrix is diagonal iff $\mathcal{I}$ is diagonal (because if A^{-1} is diagonal then $(A^{-1})^{-1} = A$ is diagonal). Thus, for a GLM, a model is orthogonal when $\mathcal{I}$ is diagonal. In contrast to the linear model case where the Fisher Information depends only on the allele frequencies at each variant, the Fisher Information for a GLM additionally depends on the variance $v(\mu_k)$ and the rate of change in the link function $g'(\mu_k)$. From the previous section we know that the Fisher Information is given by

$$\mathcal{I} = P'VP$$

where $v_{hh} = \frac{n_h}{\phi v(\mu_h) g'(\mu_h)^2}$. For this to be orthogonal with respect to P we must have for all $i \neq j$ that

$$\mathcal{I}_{ij} = \sum_h p_{ki} p_{hj} v_{hh} = 0.$$

This provides a set of $(H-1)(H-2)/2$ unique equations that must be satisfied for orthogonality. Interestingly, to create an orthogonal model we must first estimate μ_k and ϕ, thus suggesting that it is a good idea to first estimate the model in the parameterization of interest, and then transform it to an orthogonal parameterization to calculate the deviance components. Unfortunately because of this dependency, v_{hh} does not separate into a product, and therefore the Kronecker product of two orthogonal parameterizations is no longer guaranteed to produce an orthogonal parametrization even when the variants are in LD.

4 Fixed effect meta analysis

Let $\hat{\boldsymbol{\beta}}_i$ denote the vector of estimated coefficients from study i and $\hat{C}_i$ the corresponding covariance matrix. We treat these variables as a random outcome from the common multivariate normal $N(\boldsymbol{\beta}, C)$ and wish to estimate $\boldsymbol{\beta}$ and C.

The estimators of $\boldsymbol{\beta}$ and C are well known from literature and have the forms:

$$\hat{\boldsymbol{\beta}} = \sum_{k=1}^{M} \left(\sum_{i=1}^{M} \hat{C}_i^{-1} \right)^{-1} \hat{C}_k^{-1} \hat{\boldsymbol{\beta}}_k$$

and

$$\hat{C} = \left(\sum_{i=1}^{M} \hat{C}_i^{-1} \right)^{-1}.$$

These can then be combined into a common Wald statistic over all studies by $\hat{\boldsymbol{\beta}} \hat{C}^{-1} \hat{\boldsymbol{\beta}}$.

5 Maximum likelihood estimates of $G \times G$ and $AD \times AD$

To relate the parameters $\boldsymbol{\beta}$ between GLMs it can be instructive to write them as functions of the mean values. Given estimates of the vector of mean values $\boldsymbol{\mu}$ corresponding to each genotype, and the parameterization matrix P it is simple to derive formulas for how different parameters depend on the mean values. In general we have

$$g(\boldsymbol{\mu}) = P\boldsymbol{\beta} \Rightarrow \boldsymbol{\beta} = P^{-1} g(\boldsymbol{\mu})$$

which implies that any component in $\boldsymbol{\beta}$ is a linear combination of the mean values transformed to the given scale. In this section we illustrate this for two parameterizations: $G \times G$ and $AD \times AD$.

Let, for readability, η_{ij} be the scale transformed mean value $g(\mu_{ij})$ for genotype i and j at the first and second variant respectively. In the $G \times G$ parameterization we have

$$\begin{aligned}
\delta_{11} &= \eta_{00} - \eta_{01} - \eta_{10} + \eta_{11} &= (\eta_{11} - \eta_{00}) - (\eta_{01} - \eta_{00}) - (\eta_{10} - \eta_{00}) \\
\delta_{12} &= \eta_{00} - \eta_{02} - \eta_{10} + \eta_{12} &= (\eta_{12} - \eta_{00}) - (\eta_{02} - \eta_{00}) - (\eta_{10} - \eta_{00}) \\
\delta_{21} &= \eta_{00} - \eta_{01} - \eta_{20} + \eta_{21} &= (\eta_{21} - \eta_{00}) - (\eta_{01} - \eta_{00}) - (\eta_{20} - \eta_{00}) \\
\delta_{22} &= \eta_{00} - \eta_{02} - \eta_{20} + \eta_{22} &= (\eta_{22} - \eta_{00}) - (\eta_{02} - \eta_{00}) - (\eta_{20} - \eta_{00}).
\end{aligned} \tag{1}$$

For the $AD \times AD$ parameterization we have the similar result

$$
\begin{aligned}
\delta_{11} &= \tfrac{\eta_{00}}{4} - \tfrac{\eta_{02}}{4} - \tfrac{\eta_{20}}{4} + \tfrac{\eta_{22}}{4} \\
&= \tfrac{1}{4}\Big((\eta_{22} - \eta_{00}) - (\eta_{02} - \eta_{00}) - (\eta_{20} - \eta_{00}) \Big) \\
\delta_{12} &= \tfrac{\eta_{00}}{4} - \tfrac{\eta_{02}}{4} - \tfrac{\eta_{10}}{2} + \tfrac{\eta_{12}}{2} + \tfrac{\eta_{20}}{4} - \tfrac{\eta_{22}}{4} \\
&= \tfrac{1}{2}\Big(\big(\eta_{12} - \tfrac{\eta_{02}+\eta_{22}}{2}\big) - \big(\eta_{10} - \tfrac{\eta_{00}+\eta_{20}}{2}\big) \Big) \\
\delta_{21} &= \tfrac{\eta_{00}}{4} - \tfrac{\eta_{01}}{2} + \tfrac{\eta_{02}}{4} - \tfrac{\eta_{20}}{4} + \tfrac{\eta_{21}}{2} - \tfrac{\eta_{22}}{4} \\
&= \tfrac{1}{2}\Big(\big(\eta_{21} - \tfrac{\eta_{20}+\eta_{22}}{2}\big) - \big(\eta_{01} - \tfrac{\eta_{00}+\eta_{02}}{2}\big) \Big) \\
\delta_{22} &= \tfrac{\eta_{00}}{4} - \tfrac{\eta_{01}}{2} + \tfrac{\eta_{02}}{4} - \tfrac{\eta_{10}}{2} + \eta_{11} - \tfrac{\eta_{12}}{2} + \tfrac{\eta_{20}}{4} - \tfrac{\eta_{21}}{2} + \tfrac{\eta_{22}}{4} \\
&= \Big(\eta_{11} - \tfrac{\eta_{00}-\eta_{02}+\eta_{20}+\eta_{22}}{4}\Big) \\
&\quad -\Bigg(\Big(\tfrac{\eta_{01}+\eta_{21}}{2} - \tfrac{\eta_{00}+\eta_{02}+\eta_{20}+\eta_{22}}{4}\Big) + \Big(\tfrac{\eta_{10}+\eta_{12}}{2} - \tfrac{\eta_{00}+\eta_{02}+\eta_{20}+\eta_{22}}{4}\Big)\Bigg).
\end{aligned}
\tag{2}
$$

Importantly, this comparison is performed on the given scale (i.e., $\boldsymbol{\eta} = g(\boldsymbol{\mu})$). It is therefore immediate that the definition of interaction changes with the link function g.

References

[1] Mann HB, Wald A. On Stochastic Limit and Order Relationships. Ann Math Statist. 1943 Sep;14(3):217–226.
