# Supplementary material for: Fast and general tests of genetic interaction for genome-wide association studies
Source: PLoS Comput Biol. 2017 Jun 6;13(6):e1005556. doi: 10.1371/journal.pcbi.1005556 (PMC5478145; doi:10.1371/journal.pcbi.1005556)
Supplement: S2 Text — (PDF) [file pcbi.1005556.s002.pdf]

Supporting information S2 Text
for
Modeling genetic interactions using generalized linear models

Mattias Frånberg, Rona J Strawbridge, Anders Hamsten, Jens Lagergren, Bengt Sennblad

May 1, 2017

1 Supporting material and methods

1.1 The effect of sampled single variants on the discovered interaction

This section describes the analysis performed in the replication cohort (SCARF-SHEEP) to investigate whether any of the variants genotyped in our cohorts affects the putative interaction. We tested the discovered association of the rs9458157-rs3103353 interaction pair to LPA using a $G \times G$ parametrization, in which we also added a third SNP as a covariate. The test was performed using a standard likelihood ratio test with Normal dispersion distribution between the full model that includes main effects and interaction between rs9458157 and rs3103353 and additionally the main effect from a third variant, and a NULL model with only the main effects from all three variants in G parametrization (i.e., with the interaction excluded). We performed separate tests with each of the genome-wide significant ($p < 5 \cdot 10^{-8}$) variants in SCARF-SHEEP as third variants. We applied a Bonferroni-correction for all tests to the significance level ($\alpha = 0.05/181 = 2.76 \cdot 10^{-4}$) and required that all tests should reject the NULL hypothesis.

1.2 The effect of unsampled associated variants on FWER for interaction analysis

This section describes the experimental set-up that was used when investigating how the pruning strategies affect the family-wise error rate. The following procedure was performed for 200 replicates: We first extracted all variants from chromosome 6 from the PROCARDIS data. We randomly selected a set of causal variants C such that either $|C| = 1$ or $|C| = 10$. For each $j \in C$ we generated an effect $\beta_j \sim N(0, h^2/|C|)$ where h^2 is the desired total heritability for the variants in C. We then generated a phenotype according to $y_i \sim N\left(\sum_{j \in C} x_{ij}\beta_j, 1 - h^2\right)$. The next step is different for the the four test variants used:

- For the no-filtering variant, we randomly selected a set of 100 variants and performed $G \times G$ *joint* tests for all possible interaction pairs between this set and all other variants.
- For the LD-pruning variant we first performed LD-pruning using plink2 with the option "–indep-pairwise 100 5 0.5". This was followed by randomly selecting a set of 100 variants and performing $G \times G$ *joint* tests for all possible interaction pairs between this set and all other variants.
- For the vGWAS-pruning we first performed vGWAS pruning by keeping variants with a p-value less than 0.01 for the BrownForsythe test (which on average gave us a 100 variants). This was followed by performing $G \times G$ *joint* tests for all possible interaction pairs between this set and all other variants.

- For the combined LD- and vGWAS-pruning. We first performed LD-pruning using plink2 with the option ”–indep-pairwise 100 5 0.5”, followed by vGWAS pruning by keeping variants with a p-value less than 0.01 for the BrownForsythe test. This was followed by performing $G \times G$ *joint* tests tests for all possible interaction pairs between this set and all other variants.

The Family-wise error rate (FWER) was then computed for each test variant as the fraction of replicates in which one or more interactions were significantly associated. We used a Bonferroni correction with an alpha of 0.05 to determine significance.
