# Supplementary figures and images for: Fast and general tests of genetic interaction for genome-wide association studies
Source: PLoS Comput Biol. 2017 Jun 6;13(6):e1005556. doi: 10.1371/journal.pcbi.1005556 (PMC5478145; doi:10.1371/journal.pcbi.1005556)

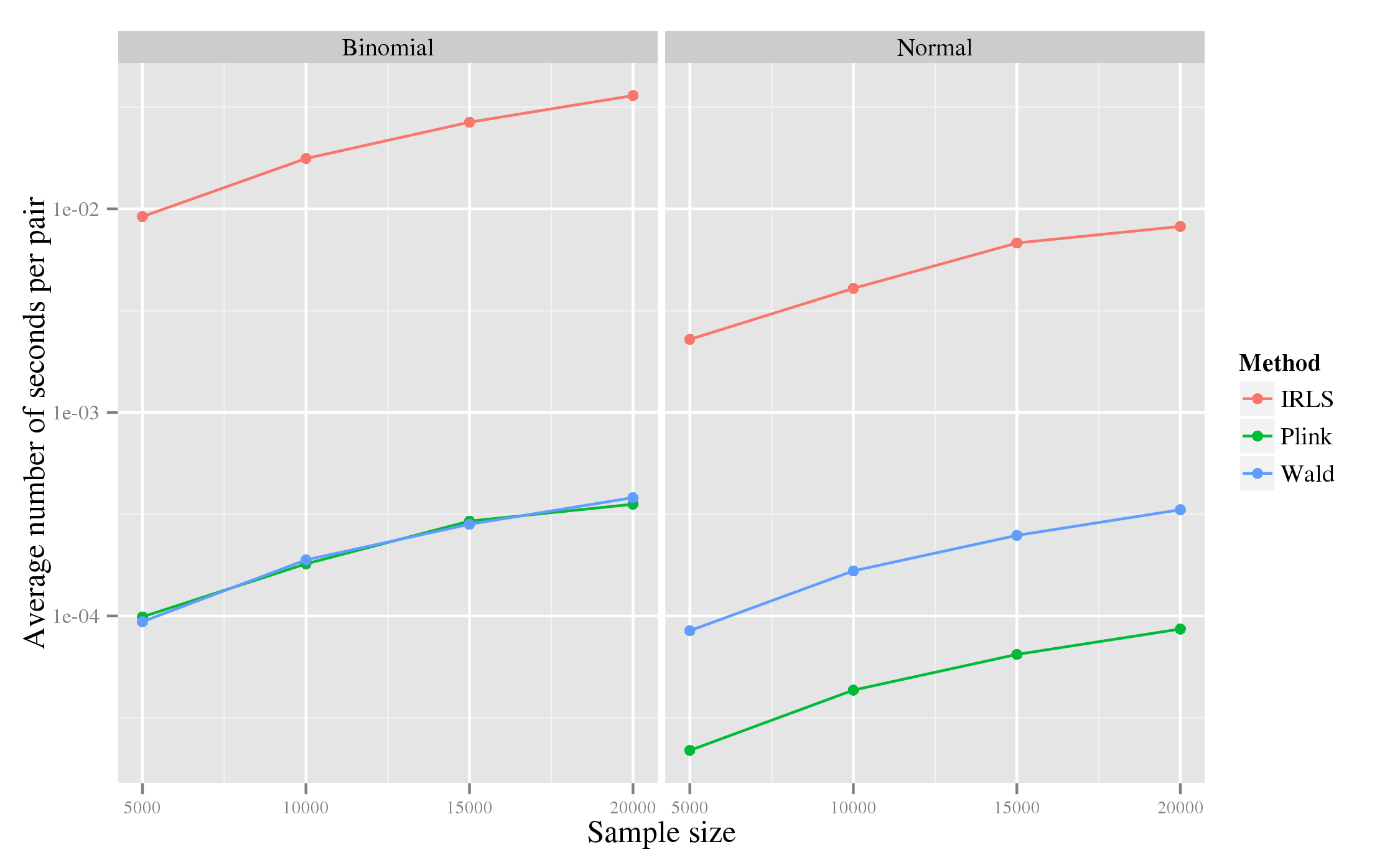

Supplement: S1 Fig — The left and right subplots are for binary and continuous phenotypes respectively. The x-axis is the total sample size, for the binary phenotype this value is cases plus controls. The y-axis is the average time required to compute the p-value per variant pair on a log-scale. The colors represent the algorithm used to compute the test statistics, “Plink” refer to the –epistasis-test in the Plink 1.90 Beta software, “Wald” refer to the Wald test, and “IRLS” refer to the likelihood ratio test implemented using the iteratively reweighed least squares algorithm. (TIFF) [file pcbi.1005556.s003.tiff]

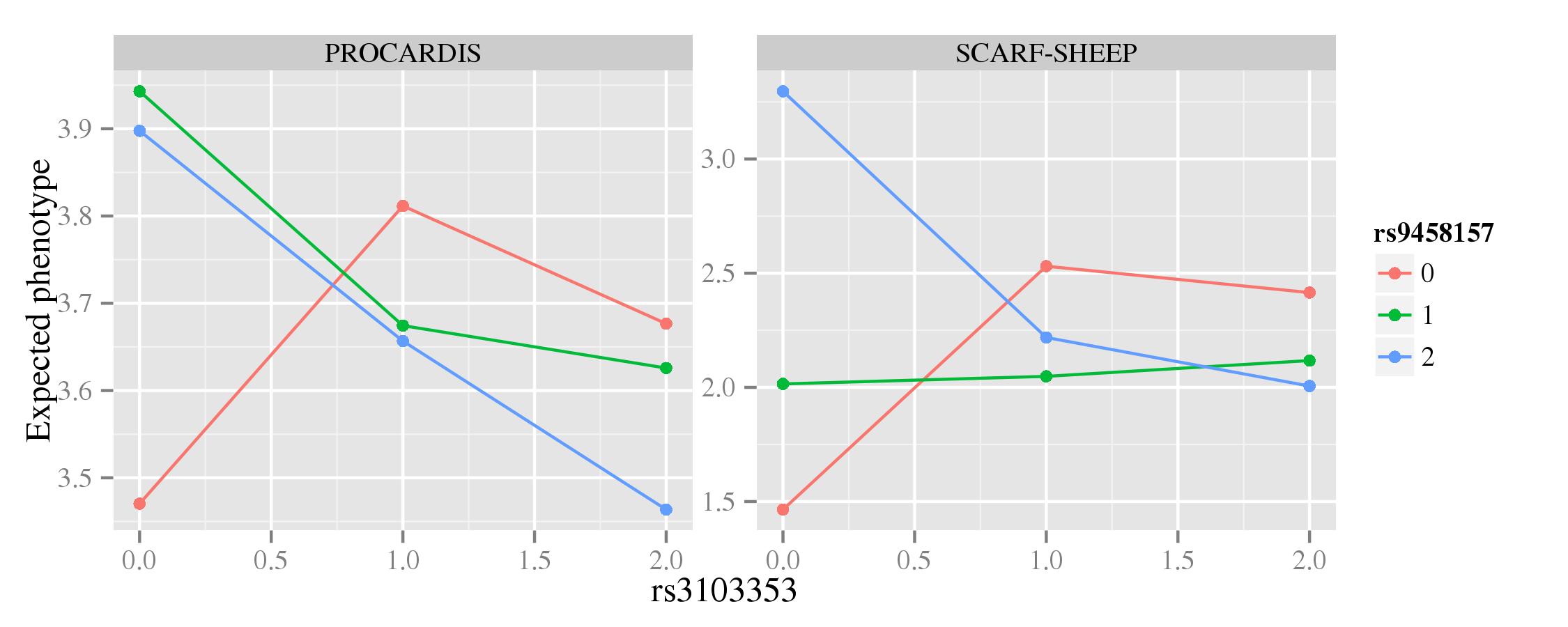

Supplement: S2 Fig — The x-axis is the number of minor alleles of the first variant. The y-axis is the expected value of the phenotype. The colors correspond to the number of minor alleles of the second variant. (TIFF) [file pcbi.1005556.s004.tiff]

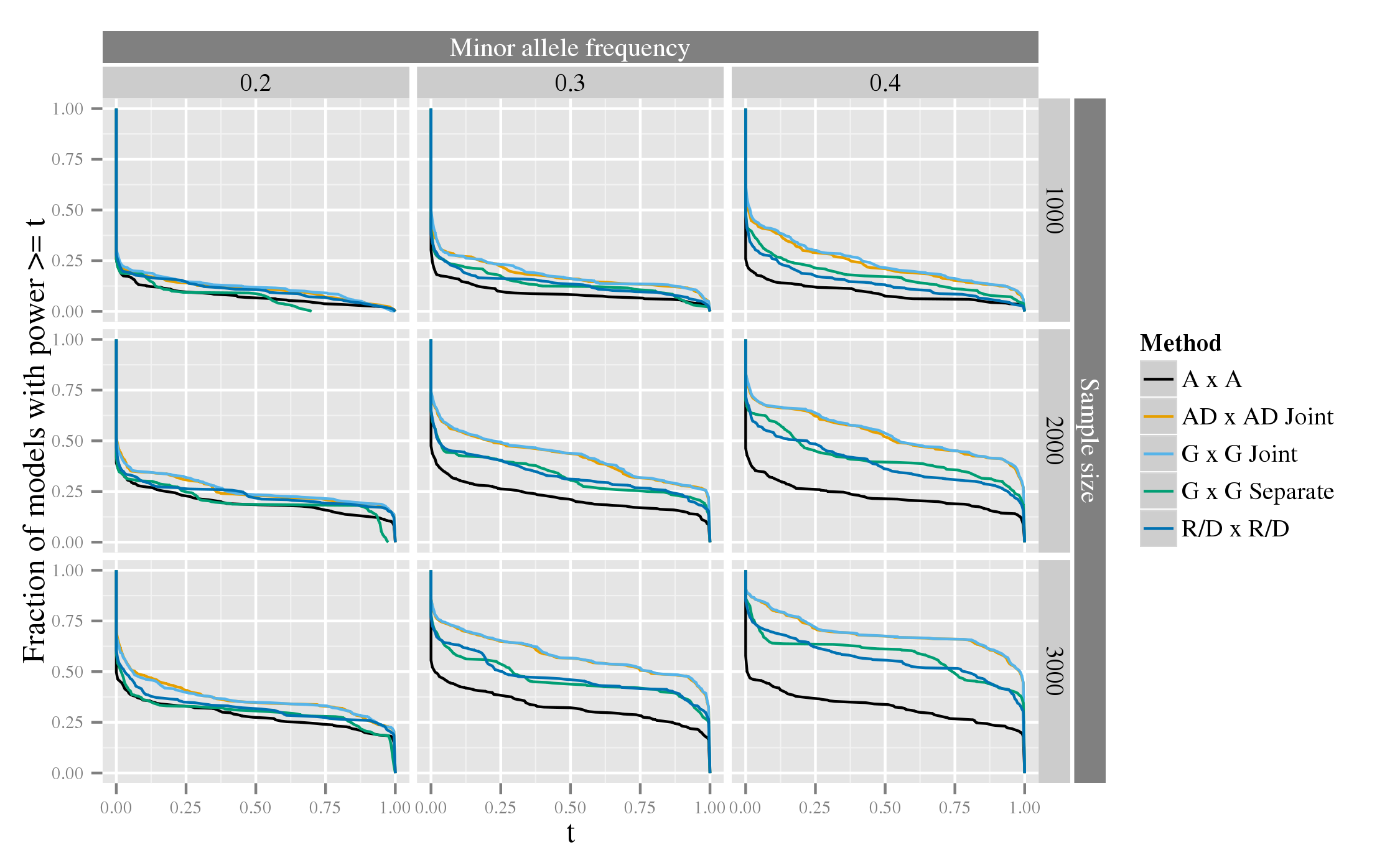

Supplement: S3 Fig — For each plot, the x-axis shows a threshold, t, for power to detect an interaction among 1012 variant pairs, and the corresponding y-axis shows the fraction of generative models, for which the analysis have a power greater than or equal to t. The rows correspond to the sample size. The columns correspond to the minor allele frequency of both variants in the pair. The line for the AD × AD joint test is often obscured by the line for the G × G joint test. (TIFF) [file pcbi.1005556.s005.tiff]

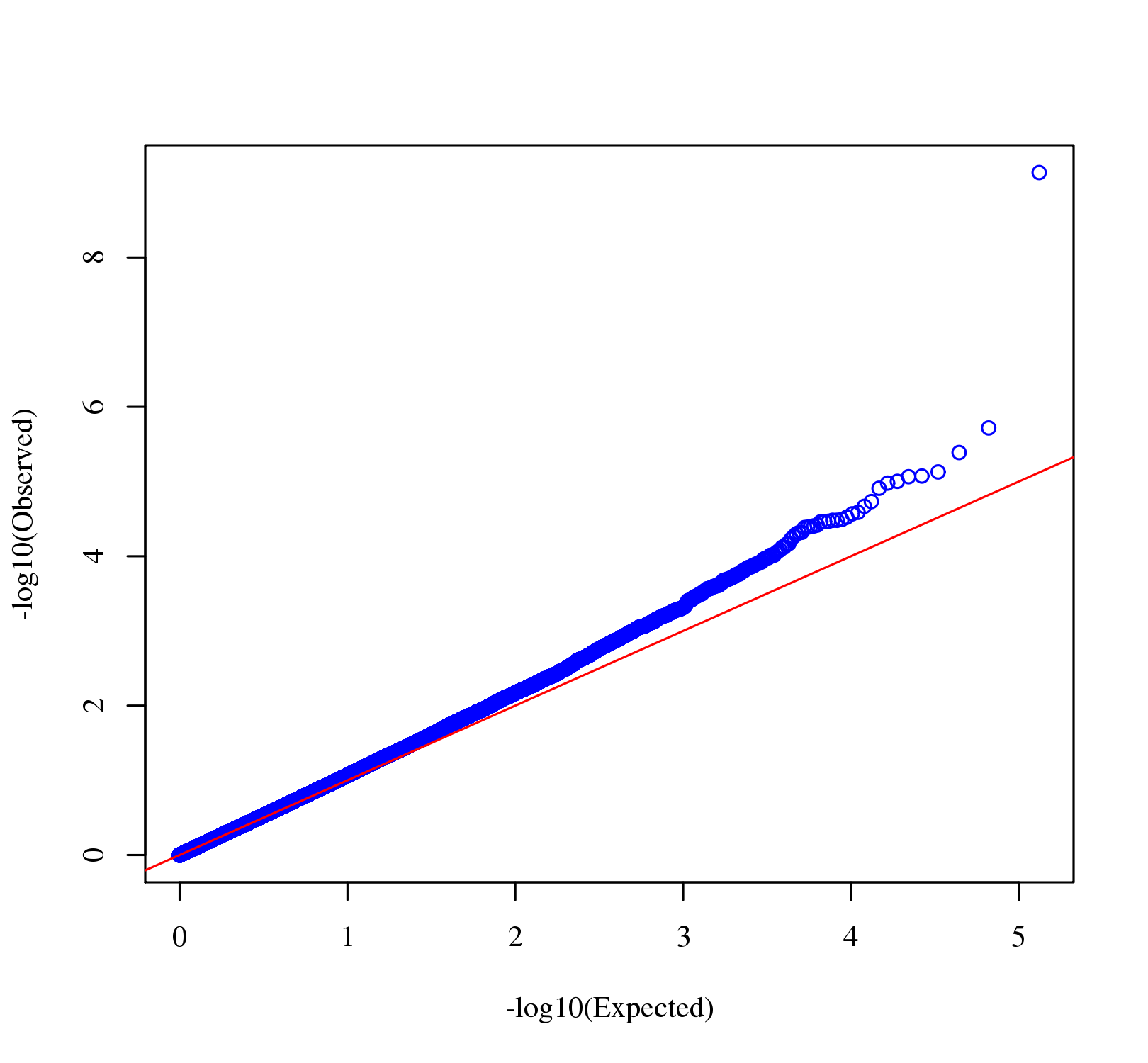

Supplement: S4 Fig — The x-axis is the expected log p-values and the y-axis is the observed log p-values. There is little deviation from the diagonal line and genomic inflation is low (λ = 1.102). (TIFF) [file pcbi.1005556.s006.tiff]

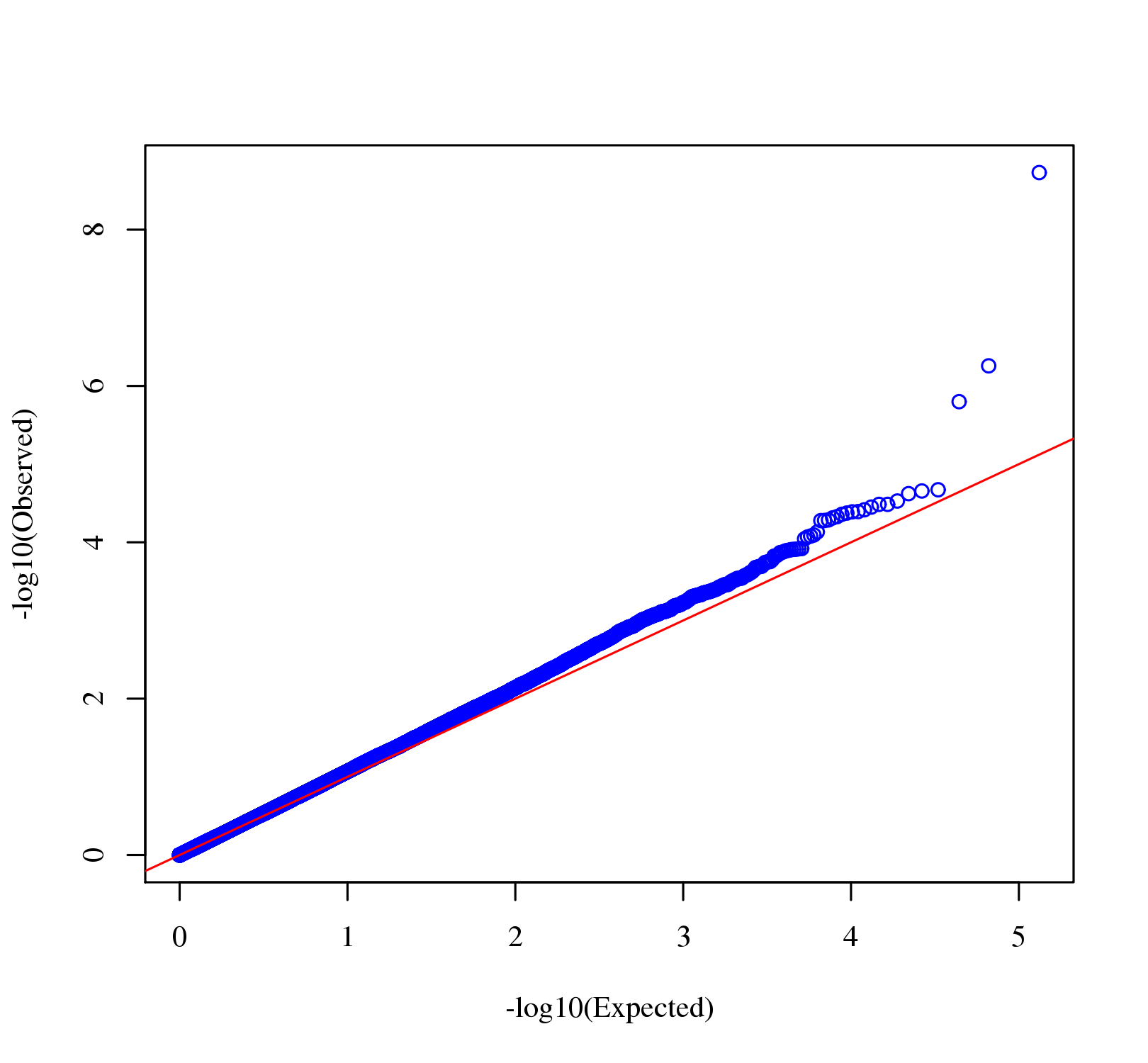

Supplement: S5 Fig — The x-axis is the expected log p-values and the y-axis is the observed log p-values. There is little deviation from the diagonal line and genomic inflation is low (λ = 1.100). (TIFF) [file pcbi.1005556.s007.tiff]

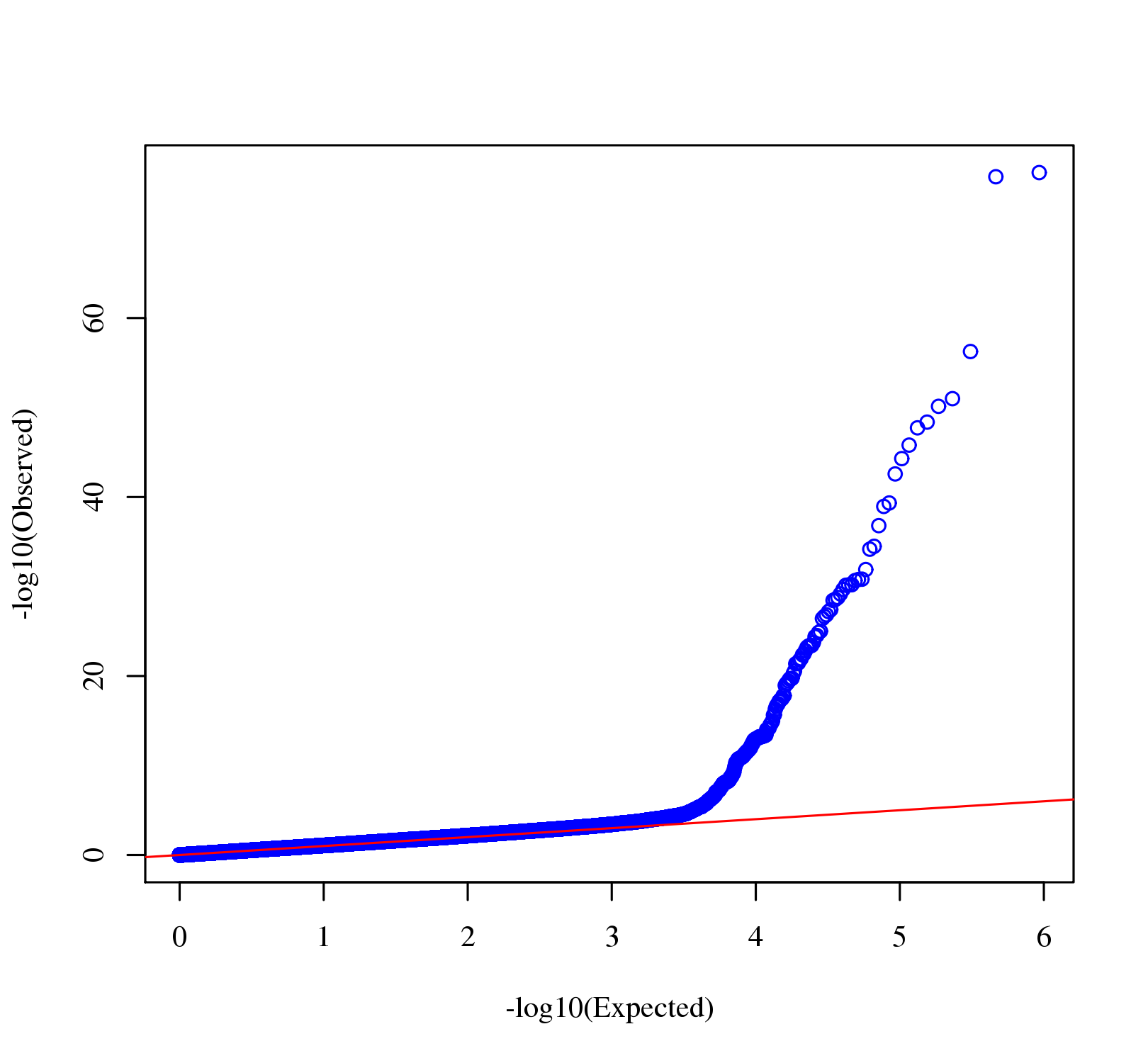

Supplement: S6 Fig — The x-axis is the expected log p-values and the y-axis is the observed log p-values. There is substantial deviation from the diagonal line; however, genomic inflation is low (λ = 1.109). (TIFF) [file pcbi.1005556.s008.tiff]

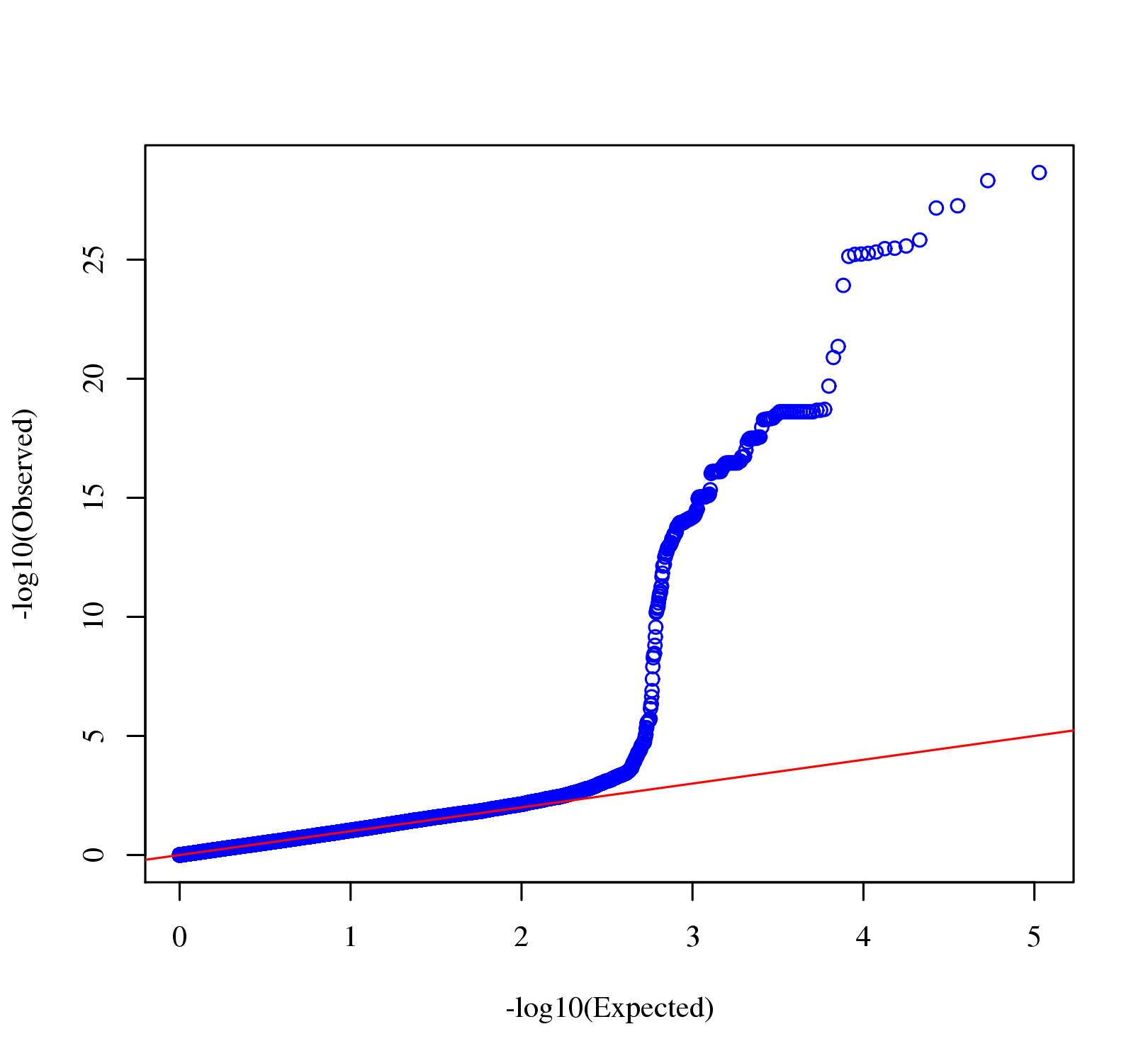

Supplement: S7 Fig — The x-axis is the expected log p-values and the y-axis is the observed log p-values. There is substantial deviation from the diagonal line; however, genomic inflation is low (λ = 1.047). (TIFF) [file pcbi.1005556.s009.tiff]

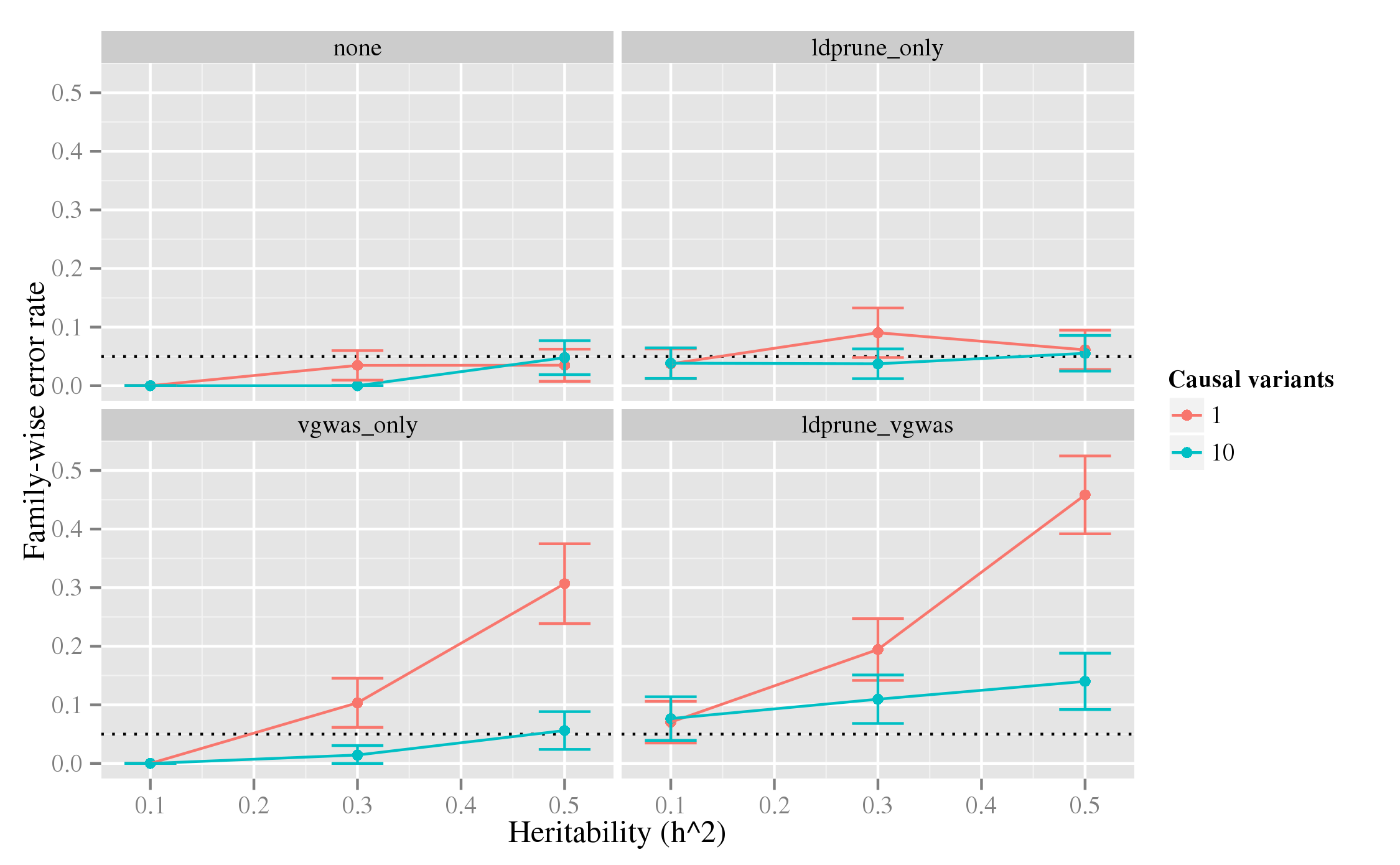

Supplement: S8 Fig — The x-axis is the total heritability of the generated variants. The y-axis is the estimated family-wise error rate. The colors correspond to the number of causal variants. The dashed line is the 0.05 FWER threshold. The error bar of each point is the 95% confidence interval of the corresponding FWER estimate. Each subplot corresponds to 4 different pruning strategies: no pruning, only ld-pruning, only vGWAS pruning, and both ld-pruning and vGWAS pruning. (TIFF) [file pcbi.1005556.s010.tiff]
