# Supplementary material for: Fast and general tests of genetic interaction for genome-wide association studies
Source: PLoS Comput Biol. 2017 Jun 6;13(6):e1005556. doi: 10.1371/journal.pcbi.1005556 (PMC5478145; doi:10.1371/journal.pcbi.1005556)
Supplement: S1 Table — The first column is the name of the link function. The second column is the form of the link function. The third column is the bias when the link function is misspecified by a small perturbation ϵ using parameterization matrix P. Here diag(x) denotes the diagonal matrix with the vector x as the diagonal. (PDF) [file pcbi.1005556.s011.pdf]

**Link function**	$g(\boldsymbol{\mu})$	**Bias**
Identity	$\boldsymbol{\mu}$	$P^{-1}\boldsymbol{\epsilon}$
Log	$\log \boldsymbol{\mu}$	$P^{-1}\mathrm{diag}(\boldsymbol{\epsilon})e^{-P\boldsymbol{\beta}}$
Logit	$\log\left(\frac{\boldsymbol{\mu}}{1-\boldsymbol{\mu}}\right)$	$P^{-1}\mathrm{diag}(\boldsymbol{\epsilon})(2+e^{-P\boldsymbol{\beta}}+e^{P\boldsymbol{\beta}})$
