# Supplementary material for: Fast and general tests of genetic interaction for genome-wide association studies
Source: PLoS Comput Biol. 2017 Jun 6;13(6):e1005556. doi: 10.1371/journal.pcbi.1005556 (PMC5478145; doi:10.1371/journal.pcbi.1005556)
Supplement: S2 Table — The first column is the type of phenotype. The second column is the name of the dispersal distribution commonly used for the corresponding phenotype type. The third column is the canonical link function. (PDF) [file pcbi.1005556.s012.pdf]

**Type of data**	**Dispersal distribution**	**Canonical link** ($g(\mu)$)
Continuous	Normal	μ
Binary	Binomial	$\log\left(\frac{\mu}{1-\mu}\right)$
Count	Poisson	$\log(\mu)$
