# Supplementary material for: Fast and general tests of genetic interaction for genome-wide association studies
Source: PLoS Comput Biol. 2017 Jun 6;13(6):e1005556. doi: 10.1371/journal.pcbi.1005556 (PMC5478145; doi:10.1371/journal.pcbi.1005556)
Supplement: S3 Table — The abbreviation ‘Enh’ stands for Enhancer and ‘Pro’ for Promoter, see the Roadmap epigenomics project for further details. (PDF) [file pcbi.1005556.s013.pdf]

rsid	Closest gene	Distance to closest gene	Location in closest gene	Epigenetic Chromatin states	Epigenetic Chromatin marks	Epigenetic DNase	eQTL
Lp(a)							
rs3103353	*SLC22A2*	0	3'UTR	FAT:15_EnhAF; VASCULAR:18_EnhAc	FAT:H3K4me1_Enh		*SLC22A3*, Skin Sun Exposed Lower leg (GTEx2015 v6)
rs9458157	*AGPAT4*	0	INTRON		BLOOD:H3K4me1_Enh; HEART:H3K4me1_Enh; VASCULAR:H3K4me1_Enh	BLOOD	
MI							
rs9830544	*NAALADL2*	0	INTRON				
rs955321	*MIR622*	314393					
rs7992498	*MIR622*	307789		FAT:16_EnhW1	FAT:H3K4me3_Pro		
rs1031097	*HS3ST1*	669312					
rs3890206	*TMX3*	362730					
rs1660241	*TCF4*	0	INTRON				*TCF4*, Whole Blood (Westra2013)
rs11666402	*PPAN*	0	INTRON	BLOOD:12_TxEnhW; LIVER:10_TxEnh5	BLOOD:H3K27ac_Enh; FAT:H3K4me1_Enh; HEART:H3K4me1_Enh; LIVER:H3K4me1_Enh; VASCULAR:H3K4me1_Enh	BLOOD	*EIF3G*, Testis (GTEx2015 v6); *ANGPTL6*, Whole Blood (Westra2013); *DNMT1*, Whole Blood (Westra2013); *P2RY11—EIF3G*, Whole Blood (Westra2013)
rs7619872	*C3orf39*	4557		FAT:7_Enh; LIVER:17_EnhW2	FAT:H3K4me1_Enh; LIVER:H3K9ac_Pro		
rs1281465	*FHIT*	70347			BLOOD:H3K9ac_Pro		
