# Supplementary material for: Fast and general tests of genetic interaction for genome-wide association studies
Source: PLoS Comput Biol. 2017 Jun 6;13(6):e1005556. doi: 10.1371/journal.pcbi.1005556 (PMC5478145; doi:10.1371/journal.pcbi.1005556)
Supplement: S5 Table — The first column is the model, the second the LD of the model, the rest of the columns are the model parameters used in the simulation (described in the context of a saturated GLM; σ is the variance of the Normal dispersion distribution). (PDF) [file pcbi.1005556.s015.pdf]

**Model**	**LD**	σ	α	β_1	β_2	γ_1	γ_2	δ_{11}	δ_{12}	δ_{21}	δ_{22}
$A \times A$	0.0	1.0	0.0	0.2	0.4	-0.1	-0.2	0.0	0.0	0.0	0.0
$A \times A$	0.8	1.0	0.0	0.2	0.4	-0.1	-0.2	0.0	0.0	0.0	0.0
$R \times A$	0.0	1.0	0.0	0.2	0.4	-0.3	-0.3	0.0	0.0	0.0	0.0
$R \times A$	0.8	1.0	0.0	0.2	0.4	-0.3	-0.3	0.0	0.0	0.0	0.0
$R \times D$	0.0	1.0	0.0	0.0	0.4	-0.3	-0.3	0.0	0.0	0.0	0.0
$R \times D$	0.8	1.0	0.0	0.0	0.4	-0.3	-0.3	0.0	0.0	0.0	0.0
