# Supplementary material for: Fast and general tests of genetic interaction for genome-wide association studies
Source: PLoS Comput Biol. 2017 Jun 6;13(6):e1005556. doi: 10.1371/journal.pcbi.1005556 (PMC5478145; doi:10.1371/journal.pcbi.1005556)
Supplement: S6 Table — The first column is the effect size used on the x-axis in the plot. The rest of the columns are the normal model parameters (described in the context of a saturated GLM; σ is the variance of the Normal dispersion distribution). (PDF) [file pcbi.1005556.s016.pdf]

**Effect size**	σ	α	β_1	β_2	γ_1	γ_2	δ_{11}	δ_{12}	δ_{21}	δ_{22}
-0.4	1.0	0.0	-0.4	-0.8	0.2	0.4	0.0	0.0	0.0	0.0
-0.3	1.0	0.0	-0.3	-0.6	0.2	0.4	0.0	0.0	0.0	0.0
-0.2	1.0	0.0	-0.2	-0.4	0.2	0.4	0.0	0.0	0.0	0.0
-0.1	1.0	0.0	-0.1	-0.2	0.2	0.4	0.0	0.0	0.0	0.0
0.0	1.0	0.0	0.0	0.0	0.2	0.4	0.0	0.0	0.0	0.0
0.1	1.0	0.0	0.1	0.2	0.2	0.4	0.0	0.0	0.0	0.0
0.2	1.0	0.0	0.2	0.4	0.2	0.4	0.0	0.0	0.0	0.0
0.3	1.0	0.0	0.3	0.6	0.2	0.4	0.0	0.0	0.0	0.0
0.4	1.0	0.0	0.4	0.8	0.2	0.4	0.0	0.0	0.0	0.0
