# Supplementary material for: Fast and general tests of genetic interaction for genome-wide association studies
Source: PLoS Comput Biol. 2017 Jun 6;13(6):e1005556. doi: 10.1371/journal.pcbi.1005556 (PMC5478145; doi:10.1371/journal.pcbi.1005556)
Supplement: S7 Table — The first column is the model, the second column is the effect size used on the x-axis of the corresponding plot, the rest of the columns are the parameters used in the simulation (described in the context of a saturated GLM; σ is the variance of the Normal dispersion distribution). (PDF) [file pcbi.1005556.s017.pdf]

**Model**	**Effect size**	σ	α	β_1	β_2	γ_1	γ_2	δ_{11}	δ_{12}	δ_{21}	δ_{22}
$A \times A$	-1.6	0.8	0.0	0.1	0.2	0.2	0.4	-0.4	-0.8	-0.8	-1.6
$A \times A$	-1.2	0.8	0.0	0.1	0.2	0.2	0.4	-0.3	-0.6	-0.6	-1.2
$A \times A$	-0.8	0.8	0.0	0.1	0.2	0.2	0.4	-0.2	-0.4	-0.4	-0.8
$A \times A$	-0.4	0.8	0.0	0.1	0.2	0.2	0.4	-0.1	-0.2	-0.2	-0.4
$A \times A$	0	0.8	0.0	0.0	0.0	0.0	0.0	0.0	0.0	0.0	0.0
$A \times A$	0.4	0.8	0.0	0.1	0.2	0.2	0.4	0.1	0.2	0.2	0.4
$A \times A$	0.8	0.8	0.0	0.1	0.2	0.2	0.4	0.2	0.4	0.4	0.8
$A \times A$	1.2	0.8	0.0	0.1	0.2	0.2	0.4	0.3	0.6	0.6	1.2
$A \times A$	1.6	0.8	0.0	0.1	0.2	0.2	0.4	0.4	0.8	0.8	1.6
$A \times A$ failed	-0.4	0.8	0.0	0.1	0.2	0.2	0.4	-1.6	-0.8	-0.8	-0.4
$A \times A$ failed	-0.3	0.8	0.0	0.1	0.2	0.2	0.4	-1.2	-0.6	-0.6	-0.3
$A \times A$ failed	-0.2	0.8	0.0	0.1	0.2	0.2	0.4	-0.8	-0.4	-0.4	-0.2
$A \times A$ failed	-0.1	0.8	0.0	0.1	0.2	0.2	0.4	-0.4	-0.2	-0.2	-0.1
$A \times A$ failed	0	0.8	0.0	0.0	0.0	0.0	0.0	0.0	0.0	0.0	0.0
$A \times A$ failed	0.1	0.8	0.0	0.1	0.2	0.2	0.4	0.4	0.2	0.2	0.1
$A \times A$ failed	0.2	0.8	0.0	0.1	0.2	0.2	0.4	0.8	0.4	0.4	0.2
$A \times A$ failed	0.3	0.8	0.0	0.1	0.2	0.2	0.4	1.2	0.6	0.6	0.3
$A \times A$ failed	0.4	0.8	0.0	0.1	0.2	0.2	0.4	1.6	0.8	0.8	0.4
$R \times D$	-0.8	0.8	0.0	0.0	0.0	0.0	0.0	0.0	-0.8	0.0	-0.8
$R \times D$	-0.6	0.8	0.0	0.0	0.0	0.0	0.0	0.0	-0.6	0.0	-0.6
$R \times D$	-0.4	0.8	0.0	0.0	0.0	0.0	0.0	0.0	-0.4	0.0	-0.4
$R \times D$	-0.2	0.8	0.0	0.0	0.0	0.0	0.0	0.0	-0.2	0.0	-0.4
$R \times D$	0	0.8	0.0	0.0	0.0	0.0	0.0	0.0	0.0	0.0	0.0
$R \times D$	0.2	0.8	0.0	0.0	0.0	0.0	0.0	0.0	0.2	0.0	0.2
$R \times D$	0.4	0.8	0.0	0.0	0.0	0.0	0.0	0.0	0.4	0.0	0.4
$R \times D$	0.6	0.8	0.0	0.0	0.0	0.0	0.0	0.0	0.6	0.0	0.6
$R \times D$	0.8	0.8	0.0	0.0	0.0	0.0	0.0	0.0	0.8	0.0	0.8
$H \times H$	-0.8	0.8	0.0	0.0	0.0	0.0	0.0	-0.8	0.0	0.0	0.0
$H \times H$	-0.6	0.8	0.0	0.0	0.0	0.0	0.0	-0.6	0.0	0.0	0.0
$H \times H$	-0.4	0.8	0.0	0.0	0.0	0.0	0.0	-0.4	0.0	0.0	0.0
$H \times H$	-0.2	0.8	0.0	0.0	0.0	0.0	0.0	-0.2	0.0	0.0	0.0
$H \times H$	0	0.8	0.0	0.0	0.0	0.0	0.0	0.0	0.0	0.0	0.0
$H \times H$	0.2	0.8	0.0	0.0	0.0	0.0	0.0	0.2	0.0	0.0	0.0
$H \times H$	0.4	0.8	0.0	0.0	0.0	0.0	0.0	0.4	0.0	0.0	0.0
$H \times H$	0.6	0.8	0.0	0.0	0.0	0.0	0.0	0.6	0.0	0.0	0.0
$H \times H$	0.8	0.8	0.0	0.0	0.0	0.0	0.0	0.8	0.0	0.0	0.0
$D \times D$	-1.6	0.8	0.0	0.0	0.0	0.0	0.0	-0.8	-0.8	-0.8	-0.8
$D \times D$	-1.2	0.8	0.0	0.0	0.0	0.0	0.0	-0.6	-0.6	-0.6	-0.6
$D \times D$	-0.8	0.8	0.0	0.0	0.0	0.0	0.0	-0.4	-0.4	-0.4	-0.4
$D \times D$	-0.4	0.8	0.0	0.0	0.0	0.0	0.0	-0.2	-0.2	-0.2	-0.2
$D \times D$	0	0.8	0.0	0.0	0.0	0.0	0.0	0.0	0.0	0.0	0.0
$D \times D$	0.4	0.8	0.0	0.0	0.0	0.0	0.0	0.2	0.2	0.2	0.2
$D \times D$	0.8	0.8	0.0	0.0	0.0	0.0	0.0	0.4	0.4	0.4	0.4
$D \times D$	1.2	0.8	0.0	0.0	0.0	0.0	0.0	0.6	0.6	0.6	0.6
$D \times D$	1.6	0.8	0.0	0.0	0.0	0.0	0.0	0.8	0.8	0.8	0.8
$D \times D$ failed	-0.4	0.8	0.0	0.0	0.0	0.0	0.0	-0.8	0.8	0.8	-0.8
$D \times D$ failed	-0.3	0.8	0.0	0.0	0.0	0.0	0.0	-0.6	0.6	0.6	-0.6
$D \times D$ failed	-0.2	0.8	0.0	0.0	0.0	0.0	0.0	-0.4	0.4	0.4	-0.4

$D \times D$ failed	-0.1	0.8	0.0	0.0	0.0	0.0	0.0	-0.2	0.2	0.2	-0.2
$D \times D$ failed	0	0.8	0.0	0.0	0.0	0.0	0.0	0.0	0.0	0.0	0.0
$D \times D$ failed	0.1	0.8	0.0	0.0	0.0	0.0	0.0	0.2	-0.2	-0.2	0.2
$D \times D$ failed	0.2	0.8	0.0	0.0	0.0	0.0	0.0	0.4	-0.4	-0.4	0.4
$D \times D$ failed	0.3	0.8	0.0	0.0	0.0	0.0	0.0	0.6	-0.6	-0.6	0.6
$D \times D$ failed	0.4	0.8	0.0	0.0	0.0	0.0	0.0	0.8	-0.8	-0.8	0.8
