# Supplementary material for: Fast and general tests of genetic interaction for genome-wide association studies
Source: PLoS Comput Biol. 2017 Jun 6;13(6):e1005556. doi: 10.1371/journal.pcbi.1005556 (PMC5478145; doi:10.1371/journal.pcbi.1005556)
Supplement: S8 Table — The first column is the variance of the normal distribution, the second column the intercept, column four to eight are the main effects. The last column denotes the sum of the interaction effect sizes, these were used to generate all possible interaction models where the mean of the non-zero effect sizes equaled this number. (PDF) [file pcbi.1005556.s018.pdf]

σ	α	β_1	β_2	γ_1	γ_2	$\delta : \sum_i \frac{\delta_i}{\lvert\{j \mid \lvert\delta_j\rvert > 0\}\rvert} =?$
1.0	0.0	0.0	0.0	0.0	0.0	0.5
1.0	0.0	0.0	0.0	0.0	0.0	0.75
1.0	0.0	0.0	0.0	0.0	0.0	1.0
1.0	0.0	0.0	0.0	0.0	0.0	1.25
