# Supplementary material for: Fast and general tests of genetic interaction for genome-wide association studies
Source: PLoS Comput Biol. 2017 Jun 6;13(6):e1005556. doi: 10.1371/journal.pcbi.1005556 (PMC5478145; doi:10.1371/journal.pcbi.1005556)
Supplement: S9 Table — For each variant pair, the p-value and sample size are given for the meta-analysis and the analyses of individual the MIGEN and the PROCARDIS cohorts. (PDF) [file pcbi.1005556.s019.pdf]

SNP 1	SNP 2	p_{meta}	N_{meta}	p_{migen}	N_{migen}	p_{proc}	N_{proc}	Closest gene 1	Closest gene 2
rs4240931	rs2452199	$3.03 \cdot 10^{-11}$	16141	$1.80 \cdot 10^{-3}$	6028	$2.64 \cdot 10^{-8}$	10113	*RNU5F-1*	*PFKP*
rs7834992	rs11704703	$8.26 \cdot 10^{-10}$	16145	$7.21 \cdot 10^{-7}$	6040	$1.90 \cdot 10^{-3}$	10105	*SAMD12*	*CRYBB2P1*
rs1149754	rs3741860	$8.68 \cdot 10^{-10}$	16139	$1.06 \cdot 10^{-3}$	6041	$2.16 \cdot 10^{-6}$	10098	*DLG5*	*CLEC9A*
rs7563687	rs4792347	$9.16 \cdot 10^{-10}$	16142	$6.55 \cdot 10^{-4}$	6037	$8.62 \cdot 10^{-6}$	10105	*ASB3*	*ELAC2*
rs1261025	rs2919389	$1.71 \cdot 10^{-9}$	15998	$2.22 \cdot 10^{-4}$	5921	$2.92 \cdot 10^{-5}$	10077	*LRRC38*	*FUT10*
